# The Highwire Ubiquitin Ligase Promotes Axonal Degeneration by Tuning Levels of Nmnat Protein

**DOI:** 10.1371/journal.pbio.1001440

**Published:** 2012-12-04

**Authors:** Xin Xiong, Yan Hao, Kan Sun, Jiaxing Li, Xia Li, Bibhudatta Mishra, Pushpanjali Soppina, Chunlai Wu, Richard I. Hume, Catherine A. Collins

**Affiliations:** 1 Department of Molecular Cellular and Developmental Biology, University of Michigan, Ann Arbor, Michigan, United States of America; 2 Neuroscience Center for Excellence, Louisiana State University Health Sciences Center, New Orleans, Louisiana, United States of America; Stanford University School of Medicine, United States of America

## Abstract

Axonal degeneration is a hallmark of many neuropathies, neurodegenerative diseases, and injuries. Here, using a *Drosophila* injury model, we have identified a highly conserved E3 ubiquitin ligase, Highwire (Hiw), as an important regulator of axonal and synaptic degeneration. Mutations in *hiw* strongly inhibit Wallerian degeneration in multiple neuron types and developmental stages. This new phenotype is mediated by a new downstream target of Hiw: the NAD+ biosynthetic enzyme nicotinamide mononucleotide adenyltransferase (Nmnat), which acts in parallel to a previously known target of Hiw, the Wallenda dileucine zipper kinase (Wnd/DLK) MAPKKK. Hiw promotes a rapid disappearance of Nmnat protein in the distal stump after injury. An increased level of Nmnat protein in *hiw* mutants is both required and sufficient to inhibit degeneration. Ectopically expressed mouse Nmnat2 is also subject to regulation by Hiw in distal axons and synapses. These findings implicate an important role for endogenous Nmnat and its regulation, via a conserved mechanism, in the initiation of axonal degeneration. Through independent regulation of Wnd/DLK, whose function is required for proximal axons to regenerate, Hiw plays a central role in coordinating both regenerative and degenerative responses to axonal injury.

## Abstract 2

Highwire, a conserved axonal E3 ubiquitin ligase, regulates the initiation of axonal degeneration after injury in *Drosophila* by regulating the levels of the NAD+ biosynthetic enzyme, Nmnat, and the Wnd kinase.

## Author Summary

Axons degenerate after injury and during neurodegenerative diseases, but we are still searching for the cellular mechanism responsible for this degeneration. Here, using a nerve crush injury assay in the fruit fly *Drosophila*, we have identified a role for a conserved molecule named Highwire (Hiw) in the initiation of axonal degeneration. Hiw is an E3 ubiquitin ligase thought to regulate the levels of specific downstream proteins by targeting their destruction. We show that Hiw promotes axonal degeneration by regulating two independent downstream targets: the Wallenda (Wnd) kinase, and the NAD+ biosynthetic enzyme nicotinamide mononucleotide adenyltransferase (Nmnat). Interestingly, Nmnat has previously been implicated in a protective role in neurons. Our findings indicate that Nmnat protein is down-regulated in axons by Hiw and that this regulation plays a critical role in the degeneration of axons and synapses. The other target, the Wnd kinase, was previously known for its role in promoting new axonal growth after injury. We propose that Hiw coordinates multiple responses to regenerate damaged neuronal circuits after injury: degeneration of the distal axon via Nmnat, and new growth of the proximal axon via Wnd.

## Introduction

Axon degeneration can be induced by a variety of insults, including injury. When an axon is transected from the cell body, the distal axon “stump” degenerates through a regulated self-destruction process called Wallerian degeneration [1]. This process appears to be actively regulated in axons; however, the endogenous cellular machinery that regulates and executes this degeneration process is poorly understood.

Previous studies have implicated a role for the ubiquitin proteasome system (UPS) in Wallerian degeneration, since inhibition of UPS leads to a delay in the early stages of degeneration [2],[3]. One explanation for this result is that the UPS mediates bulk protein degradation via the combined action of many ubiquitin ligases. However an alternative model is that one or several specific E3 ligases target the destruction of key inhibitors of the degeneration process. Here, using an in vivo assay for Wallerian degeneration in *Drosophila*, we identify an essential role for a specific E3 ubiquitin ligase in promoting Wallerian degeneration.

The ligase, known as Highwire (Hiw) in *Drosophila*, Phr1 in mice, is well known from studies in multiple model organisms for its conserved functions in regulating axonal and synaptic morphology during development [4]–[12]. We found that mutations in *hiw* strongly inhibit the initiation of Wallerian degeneration in multiple neuronal types and developmental stages. Until recently [13],[14], such a strong loss-of-function phenotype has not been reported for this process.

Mutations in *hiw* also inhibit synaptic retraction caused by cytoskeletal mutations [15]. However the finding that Hiw promotes axonal degeneration was originally perplexing, since a known target of Hiw, the Wallenda (Wnd) MAP kinase kinase kinase (also known as dileucine zipper kinase [DLK]) [16],[17], was found to promote Wallerian degeneration in mouse DRG and *Drosophila* olfactory neurons [18]. In *hiw* mutants Wnd levels are increased [9],[16],[17], however degeneration is inhibited. A partial explanation for these opposing results is that Wnd plays a protective role in some neuronal types [19],[20]. However this alone could not account for the essential role of Hiw in Wallerian degeneration of all neuron types. These findings pointed to the existence of additional targets for Hiw.

Recent studies in vertebrate cultured neurons have suggested the NAD+ synthase enzyme nicotinamide mononucleotide adenyltransferase 2 (Nmnat2) as an attractive target of post-translational regulation in axons [21]. Nmnat2 is transported in axons, where it has a short protein half-life, and neurons depleted for Nmnat2 undergo axonal degeneration [21]. Moreover, many gain-of-function studies suggest that increasing the activity of an Nmnat enzyme in axons can effectively delay Wallerian degeneration [22],[23]. The most classic example of this comes from studies of the Wallerian degeneration Slow (*WldS*) gain-of-function mutation in the *Nmnat1* locus, which causes a greater than 10-fold delay in the degeneration of injured axons [24]. However, despite the plethora of studies examining the effect of overexpressing Nmnat enzymes [23], very little is known about the role of the endogenous Nmnat enzymes in axons and how their activity may be regulated.

In contrast to the three isoforms in vertebrates, the *Drosophila* genome contains a single *nmnat* gene, for which two splice forms are annotated. *nmnat* is an essential gene, whose depletion in neurons causes neurodegeneration [25]–[27]. Here we find that Hiw and ubiquitination negatively regulate the levels of axonal Nmnat in vivo. Moreover endogenous Nmnat is required, in parallel to Wnd, for mutations in *hiw* to inhibit degeneration. By down-regulating the levels of Nmnat protein, Hiw promotes the initiation of Wallerian degeneration in axons and synapses. Moreover, through co-regulation of the Wnd/DLK kinase, whose function is required for proximal axons to initiate new axonal growth [28]–[32], Hiw coordinates both regenerative and degenerative responses to axonal injury.

## Results

### Highwire Plays an Essential Role in Wallerian Degeneration

**Figure 1:**
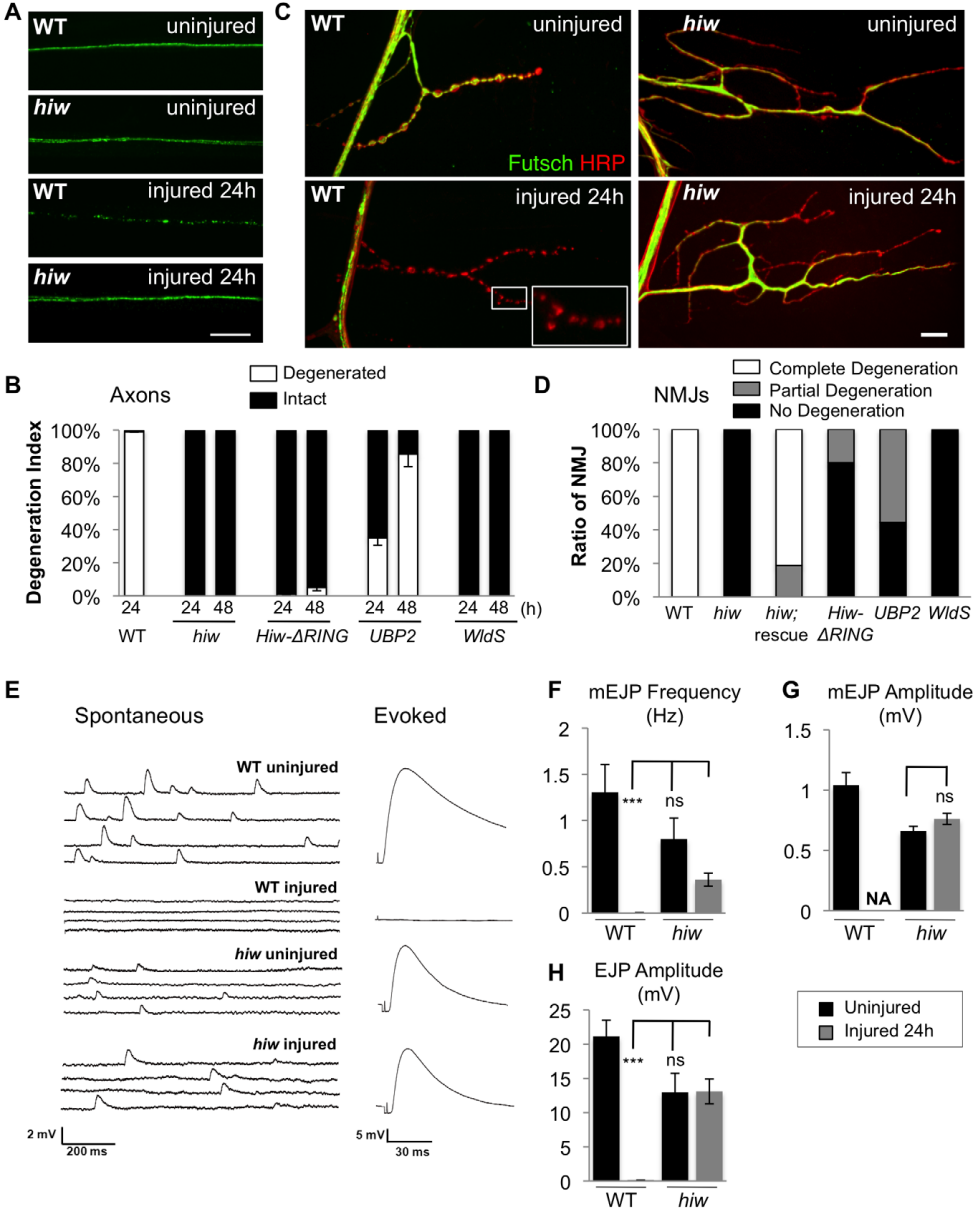
Mutations in *hiw* strongly delay Wallerian degeneration of motoneuron axons and synapses after injury. (A) In *Drosophila* third instar larvae, single axons are labeled by expression of *UAS-mCD8::GFP* with the *m12-Gal4* driver. In a wild-type (WT) background, axons distal to the injury site have completely degenerated within 24 h after nerve crush injury, however axons remain intact in the *hiw* ^Δ*N*^ mutant background. (B) Quantification of axon degeneration index in different genotypes. (See Materials and Methods and [20] for quantification methods). The degeneration index (percent degenerated) is shown with white bars, while black bars show the complementary percentage (percent intact). The genotypes are: (*UAS-mCD8::GFP/+; m12-Gal4/+*), (*hiw* ^Δ*N*^ *;UAS-mCD8::GFP/+; m12-Gal4/+*), (*UAS-mCD8::GFP/+; m12-Gal4/UAS-hiw-ΔRING*), (*UAS-mCD8::GFP/UAS-UBP2; m12-Gal4/+*), (*UAS-mCD8::GFP/+; m12-Gal4/UAS-WldS*). (C) Representative images of muscle 4 NMJs in wild-type (WT) or *hiw* ^Δ*N*^ mutants 24 h after injury. In WT animals, the presynaptic marker Futsch (green) completely disappears, while the neuronal membrane, labeled with antibodies to HRP (red), remain in discontinuous fragments. In contrast, NMJs in the *hiw* mutant (which are overgrown in an uninjured animal [8]) remain continuous and intact after injury. Of note, Futsch staining does not completely cover some synaptic branches in *hiw* mutant, but quantification of the extent of Futsch coverage (as in [20]) shows no significant difference between injured and uninjured *hiw* mutants (unpublished data). (D) Quantification of NMJ degeneration. White bars represent percentage of NMJs that have completely degenerated, defined by a complete loss of Futsch staining from the NMJ. Gray bars represent the percentage of NMJs that are partially degenerated, defined by a partial fragmentation Futsch staining and neuronal membrane (see Materials and Methods). Black bars represent the percentage of NMJs that are intact. The genotypes are: *(Canton S)*, *(hiw* ^Δ*N*^ *)*, *(hiw^ND8^, BG380-Gal4; UAS-hiw/+)*, *(BG380-Gal4;; UAS-hiw-ΔRING/+)*, *(BG380-Gal4; UAS-UBP2/+)*, *(BG380-Gal4;; UAS-WldS/+)*. (E) Representative traces of evoked and spontaneous neurotransmitter release recorded from wild-type *(Canton S)* and *hiw* mutant *(hiw* ^Δ*N*^ *)* larvae before or 24 h after injury. Calibration: 200 ms, 2 mV for spontaneous release; 30 ms, 5 mV for evoked release. (F–H) Histograms showing (F) average spontaneous miniature EJP frequency, (G) spontaneous miniature EJP amplitude, and (H) evoked EJP amplitude, either from uninjured (black bars) or injured (24 h after injury, gray bars), in *Canton S* (WT) or *hiw* ^Δ*N*^ **larvae. *n* = 10 recordings for each genotype. In WT injured larvae, only one single miniature event (amplitude 2 mV) was observed in all ten recordings. Of note, in uninjured larvae the amplitudes of evoked and miniature EJPs were smaller in *hiw* mutant, as previously reported [8]. Scale bars = 12.5 µm, error bars represent standard error; ***p<0.001; ns, not significant, *p*>0.05 in *t*-test.

We used a previously described nerve crush assay [20],[30] to study the degeneration of motoneuron and sensory neuron axons within segmental nerves in third instar *Drosophila* larvae. To quantify the degeneration of motoneuron axons, we used the *m12-Gal4* driver to label only a subset of motoneurons with *UAS-mCD8-GFP* (Figure 1A, 1B, and Materials and Methods). In wild-type (WT) animals, these axons are completely fragmented within 24 h after injury (Figure 1A) [20].

Hiw is a large, highly conserved protein thought to function as an E3 ubiquitin ligase [17],[33]. Previous studies have suggested that Hiw regulates the ability of axons to regenerate after injury [28],[30]. Here we investigated whether Hiw plays a role in degeneration after injury.

In both *hiw* null (*hiw* ^Δ*N*^ *) *and hypomorph *(hiw^ND8^)* mutant animals, axonal degeneration was strongly inhibited. Even 48 h after injury (which is the latest time that can be visualized before pupation) the distal stump of injured axons remained intact in *hiw* mutants (Figure 1A and 1B). The protection from degeneration was also recapitulated in neurons that expressed the dominant negative mutation, *hiw-*Δ*RING* (Figure 1B), but not in adjacent neurons that did not express Gal4. These results strongly suggest that Hiw performs a cell-autonomous function in promoting axonal degeneration after injury. Similarly, we found that overexpression of the de-ubiquitinating enzyme *UBP2* [34] delayed degeneration of *Drosophila* motoneuron axons and neuromuscular junctions (NMJs) (Figure 1B and 1D).

The *hiw* mutation also inhibited degeneration of the NMJ (Figure 1C). In wild-type animals, pre-synaptic proteins, such as the MAP1B homologue Futsch, disappeared completely from all NMJ boutons within 24 h after injury while the axonal membrane, detected with anti-HRP antibodies, fragmented into individual spheres (Figure 1C). In *hiw* mutants, all markers of NMJ structure remained intact (Figures 1C, 1D, and S1). Expression of *hiw* cDNA in motoneurons restored their ability to degenerate after injury (Figure 1D).

To test whether the distal stump of *hiw* mutants remained functional, NMJ synapses at muscle 6 were subjected to a standard electrophysiology recording paradigm either before or after injury (Figure 1E–1H). At 24 h after injury, wild-type NMJs were completely silent: no evoked excitatory junction potentials (EJPs) were observed (Figure 1H), and only one single spontaneous miniature event (mEJP) was observed in all ten recordings (Figure 1F). In contrast, at 24 h after injury, recordings in *hiw* mutant NMJs showed robust spontaneous mEJPs and evoked EJPs, resembling uninjured *hiw* NMJs [8]. Hence axons and synapses are functionally intact and resilient to degeneration in *hiw* mutants.

**Figure 2:**
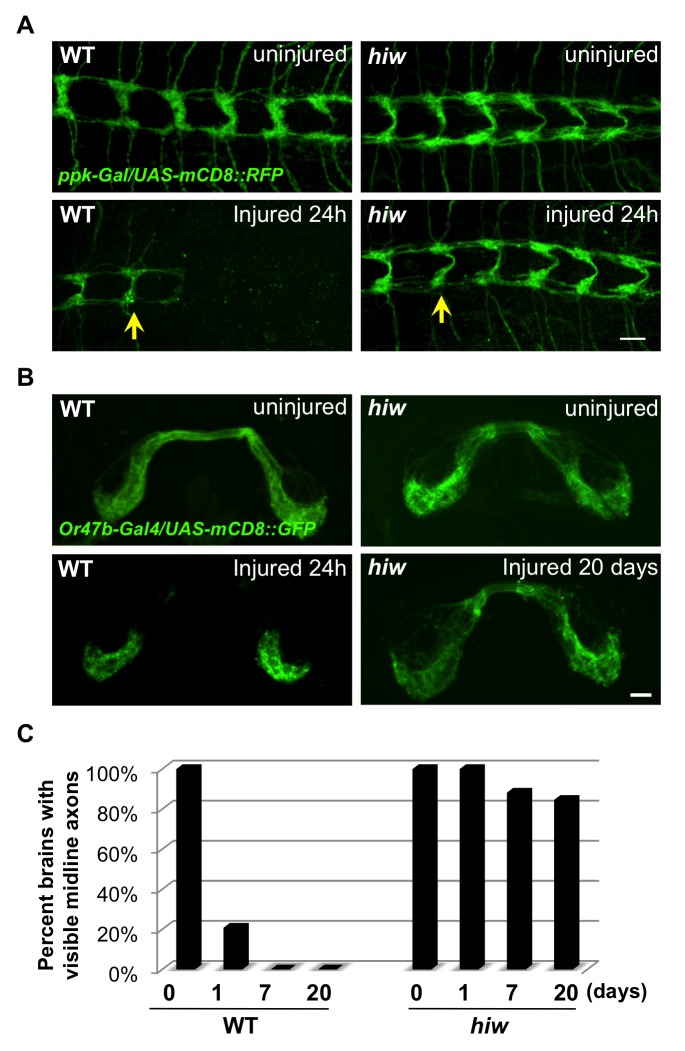
Wallerian degeneration in neurons of different neural types and developmental stages is strongly arrested in *hiw* mutants. (A) The nerve terminals of class IV sensory neurons in the ventral nerve cord, visualized by driving *UAS-mCD8::RFP* with *ppk-Gal4*, are completely degenerated and cleared within 24 h after injury in wild-type (WT) animals, however these injured axons remain intact in the *hiw* ^Δ*N*^ mutant. (Because the site of injury was in segment A2, all axons whose terminals are to the right of the yellow arrows have been injured). (B) Olfactory neuron axons in adult flies are labeled by driving expression of *UAS-mCD8::GFP* with *OR47b-Gal4*. These axons degenerate within 1 d after antenna removal in wild-type flies, however in hiw^Δ*N*^ mutants these axons remain intact even 20 d after axotomy. (C) Quantification of the percentage of animals which retain GFP-labeled commissural axons (scored as described in [2],[35]), in a time course after axotomy. Scale bars = 12.5 µm.

We then tested whether Hiw promotes axonal degeneration in other neuron types (Figure 2). The sensory neuron axons in larval segmental nerves were also injured in the nerve crush assay, and their distal axons also degenerated in a Hiw-dependent manner (Figure 2A). We then tested the role of Hiw in degeneration of adult neurons, which can be studied over a longer window of time. In wild-type animals, the distal stumps of olfactory neuron axons in the antennal lobe degenerated within 1 d after their cell bodies were removed by antennal lobe transection [2],[35]. In contrast, in *hiw* null mutants, olfactory neuron axons remained in the antennal lobe even 20 d after cell body removal (Figure 2B and 2C), which is comparable with the extent of protection by the *WldS gain-of-function *mutation [2],[35]. These dramatic phenotypes in multiple neuron types suggest that Hiw plays a fundamental role in the initiation of axonal degeneration after injury.

### The Wallenda MAPKKK Is Only Partially Required for the *highwire* Degeneration Phenotype

**Figure 3:**
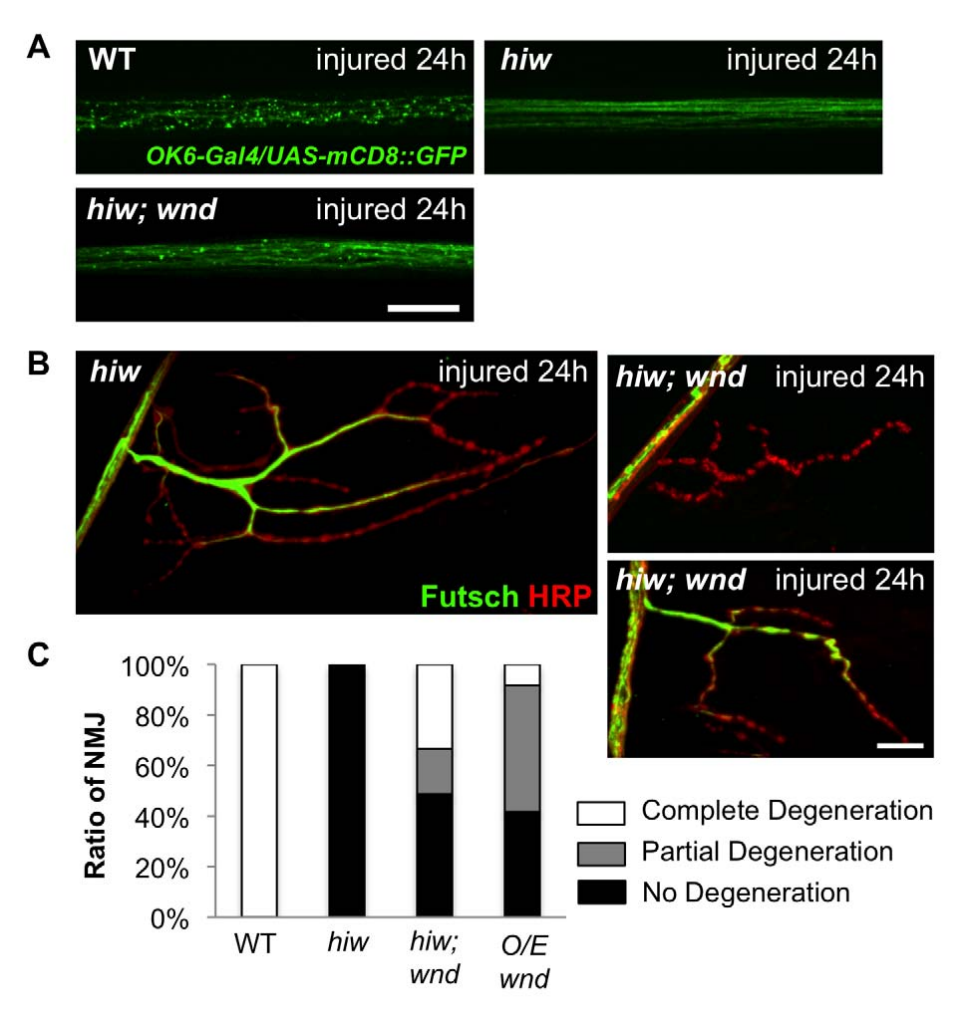
Role of the Wnd/DLK MAPKKK in Hiw-regulated degeneration. (A) *OK6-Gal4, UAS-mCD8-GFP* labeled motoneuron axons (green) are severely fragmented in wild-type (WT) axons 24 h after injury, while they remain intact in *hiw* ^Δ*N*^ mutants *(hiw* ^Δ*N*^ *; OK6-Gal4/UAS-mCD8::GFP)*. Axons in *hiw; wnd* double mutants (*hiw* ^Δ*N*^ *; OK6-Gal4/UAS-mCD8-GFP; wnd^1^/wnd^2^*) are only mildly fragmented 24 h after injury, implying that mutation of *wnd* only partially suppressed the *hiw* mutant degeneration phenotype. (B) Representative muscle 4 NMJs labeled by immunostaining for Futsch (green) and HRP (neuronal membrane, red) in *hiw* ^Δ*N*^ mutants or *hiw; wnd* double mutants *(hiw* ^Δ*N*^ *;;wnd^1^/wnd^2^)*. At 24 h after injury, NMJs have completely degenerated in wild-type (Figure 1) but are intact in *hiw* mutants. In *hiw;wnd* double mutants, some NMJs have completely degenerated (upper panel), while others remain intact (lower panel). (C) Quantification of the percentage of NMJs that are completely degenerated, partially degenerated, or intact (see Materials and Methods) for the following genotypes: *(Canton S), (hiw* ^Δ*N*^ *), (hiw* ^Δ*N*^ *;;wnd^1^/wnd^2^), (BG380-Gal4; UAS-wnd/+)*. Scale bars = 12.5 µm.

To understand the mechanism for Hiw in Wallerian degeneration we first considered a previously characterized target of Hiw regulation, the Wnd/DLK kinase. A previous study in mouse DRG and *Drosophila* olfactory neurons found that degeneration is delayed in *wnd(dlk)* mutants [18]. However, in larval motorneurons, we found the opposite result, since mutations in *hiw* lead to increased levels of Wnd kinase in axons [16], and overexpression of *wnd* in motoneuron axons can delay Wallerian degeneration [20]. Consistent with Wnd playing a protective role against degeneration downstream of Hiw, the protection from degeneration in *hiw* mutants was suppressed in *hiw; wnd* double mutants, although the suppression was only partial (Figure 3). In contrast, the synaptic overgrowth and overbranching phenotype in *hiw* mutants was completely suppressed in the *hiw;wnd* double mutants [16]. We also noticed that while *hiw* mutations inhibited degeneration in multiple neuron types, overexpression of *wnd* did not protect olfactory neuron and sensory neuron axons from degeneration [20]. Hence the degeneration phenotype for *hiw* mutants could not be accounted for by Wnd alone. This suggested the existence of additional downstream effectors of Hiw during axonal degeneration.

### Nmnat Is a Downstream Target of Highwire during Wallerian Degeneration

A well-known and intensively studied negative regulator of Wallerian degeneration is Nmnat [23]. An increased activity of this enzyme, first discovered in the *WldS* mutation, can strongly inhibit degeneration after injury [36]. This gain-of-function phenotype for *nmnat* bears a striking resemblance to the *hiw* loss-of-function phenotype in its ability to delay the onset of Wallerian degeneration.

**Figure 4:**
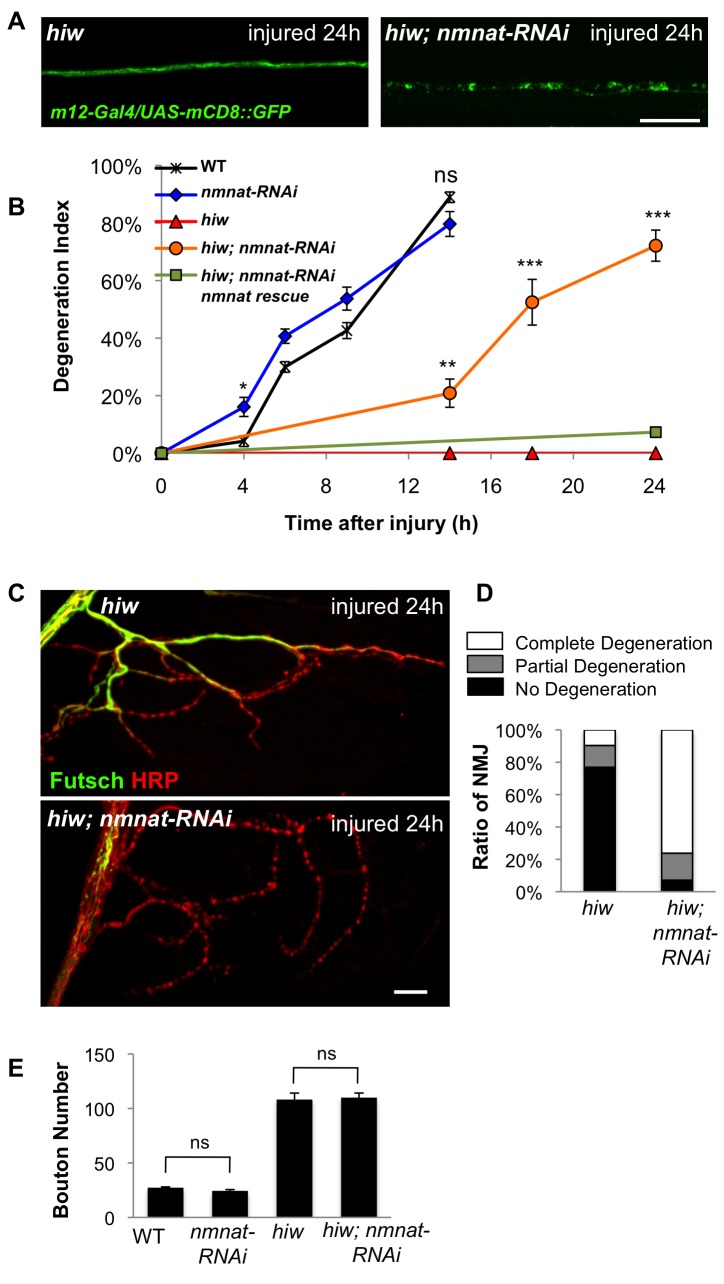
Regulation of Wallerian degeneration by Hiw depends upon endogenous Nmnat (A) Nmnat is required for the protective phenotype of *hiw. m12-Gal4*, UAS-*mCD8::GFP* labeled axons (green) 24 h after injury in animals either mutant for *hiw* (*hiw^ND8^,UAS-Dcr2; UAS-mCD8::GFP/+; m12-Gal4/+*) or mutant for in *hiw* mutant and depleted for *nmnat* by RNA interference *(hiw^ND8^, UAS-Dcr2; UAS-mCD8::GFP/UAS-nmnat-RNAi; m12-Gal4/+)*. (B) Degeneration index for the *m12-Gal4, UAS-mCD8::GFP* labeled single axons at different time points after injury in the following genotypes: *(UAS-Dcr2; UAS-mCD8::GFP/+; m12-Gal4/+), (UAS-Dcr2; UAS-mCD8-GFP/UAS-nmnat-RNAi; m12-Gal4/+), (hiw^ND8^, UAS-Dcr2; UAS-mCD8::GFP/+; m12-Gal4/+), (hiw^ND8^, UAS-Dcr2; UAS-mCD8::GFP/UAS-nmnat-RNAi; m12-Gal4/+), (hiw^ND8^, BG380-Gal4; UAS-nmnat/UAS-nmnat-RNAi; m12-Gal4,UAS-mCD8::GFP/+)*. RNAi depletion of *nmnat* alone only modestly affects the rate of degeneration (compare blue to black); however, it strongly inhibits the protection observed in the *hiw* mutant (compare orange to red). (C) Representative images of NMJs at muscle 4 24 h after injury in *hiw* mutants *(hiw^ND8^, BG380-Gal4; UAS-Dcr2/+)* or *hiw* mutants depleted for *nmnat* in neurons *(hiw^ND8^, BG380-Gal4; UAS-Dcr2/UAS-nmnat-RNAi)*. Futsch staining in green labels cytoskeleton structure and HRP staining in red labels neuronal membrane. (D) Quantification of the percentage of NMJs that are completely degenerated, partially degenerated or intact in the above genotypes. (E) Quantification of average bouton numbers per NMJ at muscle 4 in the following genotypes: (*BG380-Gal4, UAS-Dcr2), (BG380-Gal4, UAS-Dcr2; UAS-nmnat-RNAi/+), (hiw^ND8^,BG380-Gal4; UAS-Dcr2/+), (hiw^ND8^, BG380-Gal4; UAS-Dcr2/UAS-nmnat-RNAi)*. Scale bars = 12.5 µm, error bars represent standard error; *p<0.05; **p<0.01; ***p<0.001; ns, not significant, *p*>0.05 in *t*-test.

There is only one *nmnat* gene in *Drosophila* and it has been shown to be required for neural integrity [25]–[27]. To disrupt expression of this essential gene post-embryonically, we used the Gal4/UAS system to express double-stranded RNA [37] targeting *nmnat*, (*UAS-nmnat-RNAi*), in neurons. Immunostaining with an anti-Nmnat antibody [25] indicated that the knockdown of Nmnat was effective (Figure S2A); however, it was unlikely to be complete, since neuronal clones that are homozygous mutant for *Nmnat* undergo spontaneous degeneration in uninjured animals [25],[26]. In contrast, RNAi-mediated knockdown of *nmnat* in larva motoneurons did not affect the development or stability of axons and synapses (Figure S2B), and only modestly affected the time course of degeneration after injury (Figure 4B). However knockdown of *nmnat* strongly suppressed the *hiw* protective phenotype, both in axons (Figure 4A and 4B) and NMJ synapses (Figure 4C and 4D). Similarly, reduction of Nmnat also suppressed the protection from degeneration caused by overexpression of *UBP2* (Figure S3). These results suggest that Nmnat function is an important component of Hiw's role in the degeneration process. Interestingly the NMJ synaptic overgrowth phenotype of the *hiw* mutants was not suppressed by RNAi knockdown of *nmnat* (Figure 4C and 4E). This implies that Hiw regulates synaptic morphology independently of Nmnat function, or at least through a mechanism that is less sensitive to Nmnat function than degeneration. In contrast, Wnd is required for synaptic overgrowth in *hiw* mutants, and data presented below suggest that Nmnat and Wnd function independently.

### Wallenda and Nmnat Function in Parallel Downstream of Highwire

**Figure 5:**
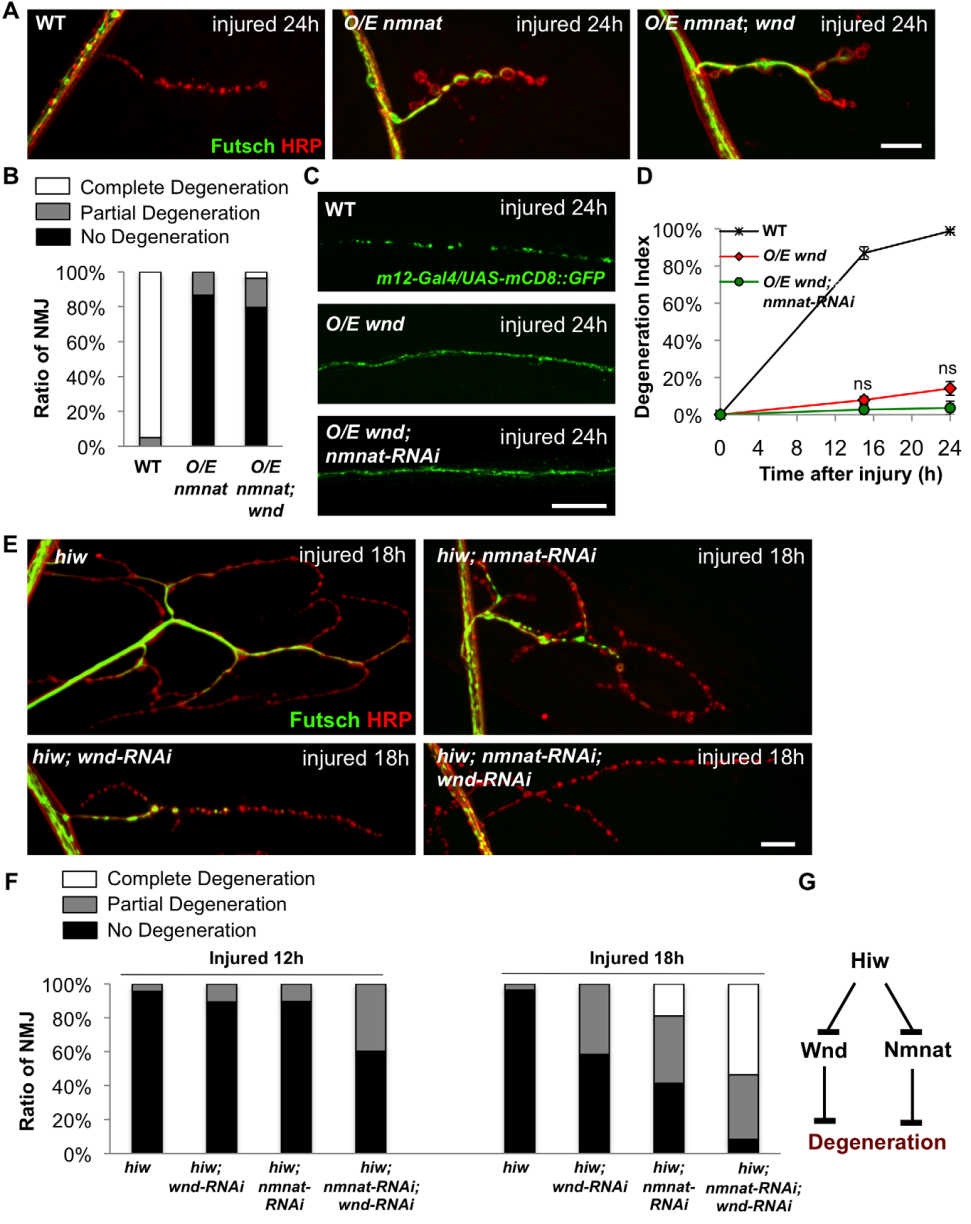
Wnd/DLK and Nmnat protect axons through parallel mechanisms downstream of Hiw. (A) Representative muscle 4 NMJs at 24 h after injury immunostained for Futsch (green) and HRP (neuronal membrane, red) for the following genotypes: WT *(Canton S)*, neuronally overexpressed *nmnat (BG380-Gal4; UAS-HA-nmnat/+)*, or overexpressed *nmnat* in a *wnd* mutant background *(BG380-Gal4; UAS-HA-nmnat/+; wnd^1^/wnd^2^)*. Overexpression of *nmnat* protected NMJs from degeneration and this protection was not compromised by mutations in *wnd*. (B) Quantification of NMJ degeneration in the above genotypes. *(C) UAS-mCD8::GFP/+; m12-Gal4/+* labeled singles axons (green) 24 h after injury in WT *(UAS-Dcr2; UAS-mCD8::GFP/+; m12-Gal4/+)*, when overexpressing *wnd (UAS-Dcr2; UAS-mCD8-GFP/+; m12-Gal4/UAS-wnd)*, or when overexpressing *wnd* in conjunction with *nmnat RNAi (UAS-Dcr2; UAS-mCD8-GFP/UAS-nmnat-RNAi; m12-Gal4/UAS-wnd)*. Reducing Nmnat levels by this method had no effect upon the protection caused by overexpression of *wnd*. (D) Degeneration index of the *m12-Gal4, UAS-mCD8::GFP* labeled single axons at different time points after injury in the above genotypes. (E) Representative images of NMJs at muscle 4 18 h after injury stained for Futsch (green) and HRP (red) in the following genotypes: *(hiw^ND8^,BG380-Gal4; UAS-Dcr2/+), (hiw^ND8^, BG380-Gal4;UAS-Dcr2/+;UAS-wnd-RNAi/+), (hiw^ND8^, BG380-Gal4; UAS-Dcr2/UAS-nmnat-RNAi), (hiw^ND8^, BG380-Gal4; UAS-Dcr2/UAS-nmnat-RNAi; UAS-wnd-RNAi/+)*. (F) Quantification of the percentage of NMJs that are completely degenerated, partially degenerated, or intact at 12 h or 18 h after injury, for the genotypes described above. (G) Model: Wnd and Nmnat inhibit axonal degeneration through independent pathways downstream of Hiw. Scale bars = 12. 5 µm, error bars represent standard error; *p<0.05; ns, not significant, *p*>0.05 in *t*-test.

To further probe the relationship between Wnd and Nmnat, we conducted genetic epistasis analysis. Overexpression (*O/E*) of either *wnd* or *nmnat* cDNA can delay Wallerian degeneration in *Drosophila* motoneurons (Figure 5A–5D), so we tested whether the phenotype of *O/E nmnat* required *wnd*, and vice versa, whether the phenotype of *O/E wnd* required *nmnat*.

We found that disruption of *wnd* function had no effect upon the protection from degeneration by *O/E nmnat* (Figure 5A and 5B). For the converse experiment, we tested whether knockdown of *nmnat* by expression of UAS-*nmnant-RNAi* affected the protection by *O/E wnd* (Figure 5C and 5D). While this method for disrupting Nmnat suppressed the *hiw* degeneration phenotype (Figure 4), it had no effect upon the *O/E wnd* phenotype (Figure 5C and 5D). These observations suggest that Nmnat and Wnd protect axons from degeneration through independent mechanisms.

We then tested whether knockdown of *nmnat* and *wnd* by RNA interference had additive effects in suppressing the *hiw* degeneration phenotype (Figure 5E and 5F). Since *nmnat*-*RNAi* rescues the *hiw* phenotype very strongly on its own at 24 h after injury, we assayed earlier time points, 12 and 18 h after injury, for additive effects with *wnd-RNAi*. Expression of *wnd-RNAi* alone in the *hiw* mutant background caused 42% of the NMJs to degenerate (including complete degeneration and partial degeneration) within 18 h of injury, while expression of *nmnat-RNAi* alone caused 59% of the *hiw* mutant NMJs to degenerate at this time point. Combined knockdown of both *nmnat* and *wnd* led to a nearly complete suppression of the *hiw* degeneration phenotype, with 92% of the NMJs degenerating (Figure 5E and 5F). Together, these results suggest that Wnd and Nmnat function independently downstream of Hiw in the Wallerian degeneration process (Figure 5G).

### Highwire Regulates the Levels of Nmnat Protein

**Figure 6:**
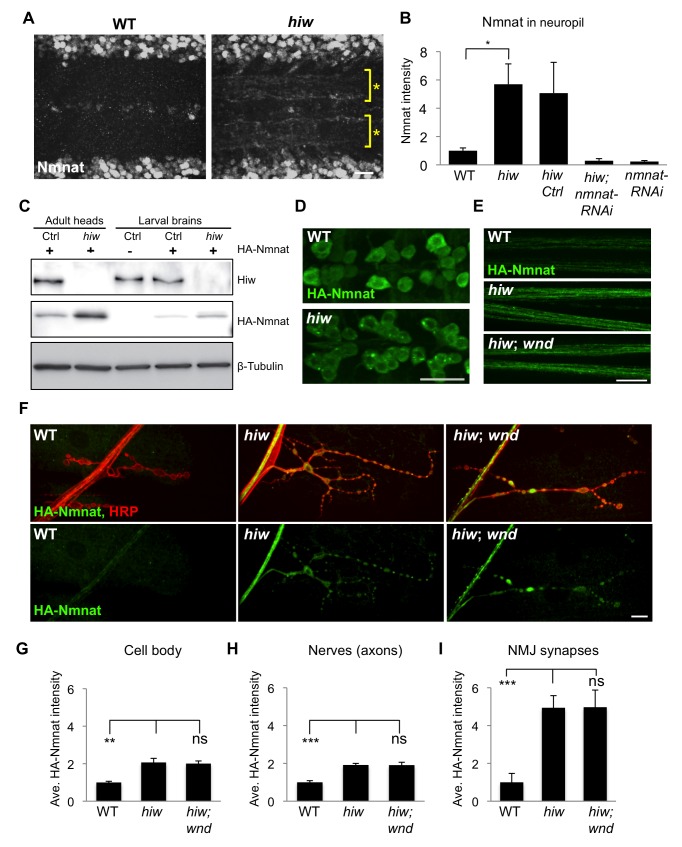
Hiw negatively regulates the levels of Nmnat protein in axons and synapses. (A) Hiw regulates endogenous Nmnat protein in neuropil. In *hiw* ^Δ*N*^ mutants, Nmnat protein can be detected within the neuropil of the ventral nerve cord, denoted with asterisks. This area of the nerve cord is devoid of cell bodies and enriched in neurites and synapses. (B) Quantification (relative levels) of Nmnat staining in neuropil, for WT (*w118*), *hiw* mutant (*hiw* ^Δ*N*^), *hiw*,*Ctrl* (*hiw* ^Δ*N*^, *BG380-Gal4*), *hiw*, *nmnat-RNAi* (*hiw* ^Δ*N*^, *BG380-Gal4*, *UAS-nmnat-RNAi*), and *nmnat-RNAi* (*BG380-Gal4*, *UAS-nmnat-RNAi*). See Materials and Methods. (C) Western blot with adult heads or larval brains to compare total protein levels of HA-Nmnat in wild-type (ctrl) and *hiw* mutant backgrounds. The *UAS-HA::nmnat* transgene is expressed in neurons with the *BG380-Gal4* driver, and males are used for all experiments. (D–F) The UAS-HA-Nmnat transgene was expressed in motoneurons with the OK6-Gal4 driver, in wild-type *(OK6-Gal4/UAS-HA::nmnat), hiw* mutant (*hiw* ^Δ*N*^;*OK6-Gal4/UAS-HA::nmnat*) and *hiw; wnd* double mutant *(hiw* ^Δ*N*^ *;OK6-Gal4/UAS-HA::nmnat;wnd^1^/wnd* ^2^ *)* backgrounds. HA-Nmnat protein is detected by immunostaining for HA. (D) Representative images of HA-Nmnat in motoneuron cell bodies, (E) segmental (peripheral) nerves, and (F) NMJ synapses, stained for anti-HA (green) and HRP (neuronal membrane, red). (G–I) Quantification of the average HA-Nmnat intensity for the above genotypes in (G) cell bodies, (H) segmental nerves, and (I) NMJ synapses at muscle 4. See Materials and Methods for details about quantification methods. In *hiw* mutants, Nmnat intensity is increased, particularly at NMJ synapses. Loss of *wnd*, in *hiw;wnd* double mutants, has no effect upon this increase. Scale bars = 12.5 µm, error bars represent standard error; *p<0.05; ***p<0.001; ns, not significant, *p*>0.05 in *t*-test.

Hiw and its homologues are known to function within an E3 ubiquitin ligase complex [17],[33],[38]–[41]. An attractive hypothesis is that Hiw promotes ubiquitination and protein turnover of endogenous Nmnat protein. Consistent with this hypothesis, we found that knockdown of *nmnat* suppressed the protection from degeneration caused by overexpression of the de-ubiquitinating enzyme *UBP2* (Figure S3). We therefore asked whether mutation in *hiw* leads to an increase in the levels of Nmnat protein. Most strikingly, we noticed an appearance of Nmnat protein in the synapse and neurite-rich neuropil of *hiw* mutants, which was not detectable in a wild-type background (Figure 6A and 6B). We also observed complex changes in the distribution of Nmnat in neuronal nuclei and glia (Figure S2).

To test whether Hiw regulates Nmnat in neurons via a post-transcriptional mechanism, we drove expression of transgenic HA-tagged *nmnat* cDNA in neurons via an ectopic Gal4/UAS promoter. In *hiw* mutants, the total level of HA-Nmnat protein, as detected on Western blots, increased in both larval brains (3.1±0.6-fold) and adult heads (5.2±1.1-fold) (Figure 6C). By immunocytochemistry, the HA-Nmnat protein (which represents a splice form that lacks the nuclear localization sequence) could readily be detected in motoneuron cell bodies (Figure 6D and 6G) and axons within segmental nerves (Figure 6E and 6H), but is barely detectable at NMJ synapses (Figure 6F and 6I). In *hiw* mutants, the levels of HA-Nmnat increased in all compartments, however the 5-fold increase quantified at NMJ synapses was most striking (Figure 6G–6I). The increase in Nmnat protein levels remained in the *hiw;wnd* double mutant background (Figure 6E–6I), adding further support to the model that Hiw regulates Nmnat protein independently of Wnd.

### Highwire Regulates Nmnat Via Ubiquitination

**Figure 7:**
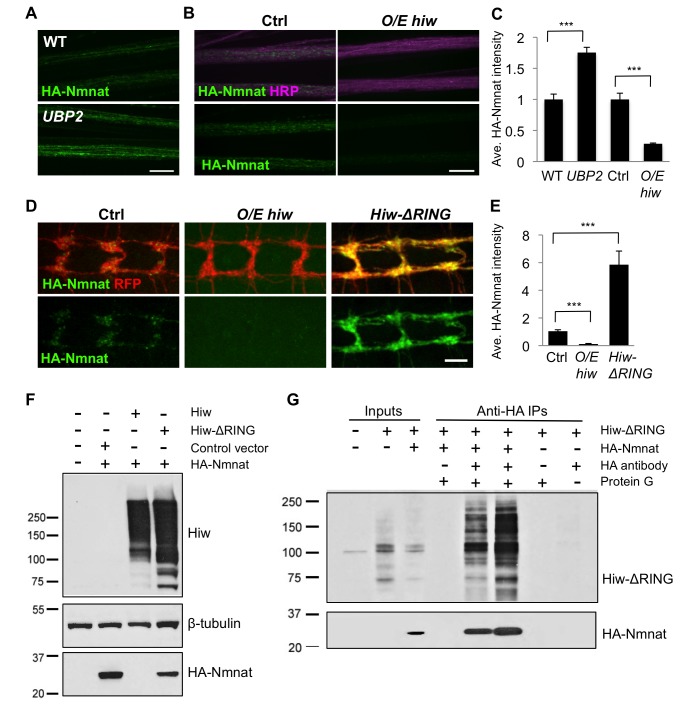
Hiw and ubiquitination down-regulate Nmnat protein. (A) The UAS-HA-nmnat transgene is expressed in motoneurons with *OK6-Gal4* driver in a wild-type (WT)_ genetic background (*OK6-Gal4,UAS-HA::nmnat/+*) or when the yeast deubiquitinase *UBP2* is co-expressed (*OK6-Gal4,UAS-HA::nmnat*/UAS*-UBP2*). Segmental nerves stained with anti-HA antibody (green). (B) Transgenic *HA-nmnat* is co-expressed with a control *UAS*- construct *(BG380-Gal4; UAS-HA::nmnat/+; UAS-nls::GFP/+)* or with the full-length *hiw* cDNA *(BG380-Gal4; UAS-HA::nmnat/UAS-hiw)*. Segmental nerves stained for HA (green) and HRP (neuronal membrane, violet). (C) Quantification of the average HA-Nmnat intensity in segmental nerves in (A) and (B). The average intensity is normalized to the control for each experimental group. (D) Distal axons and axon terminals of *ppk-Gal4,UAS-mCD8::RFP* labeled sensory neurons stained for HA (green) and RFP (red). The UAS-HA-Nmnat transgene is co-expressed with a control *UAS*- construct *(UAS-HA::nmnat/+; ppk-Gal4,UAS-mCD8::RFP/UAS-nls::GFP)*, or the full-length *hiw* cDNA *(UAS-HA::nmnat/UAS-hiw; ppk-Gal4,UAS-mCD8::RFP/+)*, or a dominant negative *hiw* transgene mutated for conserved cysteines in the RING domain [67] *(UAS-HA::nmnat/+; ppk-Gal4,UAS-mCD8::RFP/UAS-hiw-*Δ*RING)*. (E) Quantification of the average HA-Nmnat intensity in the sensory neuron axon terminals for the above genotypes. See Materials and Methods for the quantification method. (F) Hiw can down-regulate Nmnat protein in S2R+ cells. S2R+ cells were co-transfected with pUAST-HA::Nmnat, and either pUAST-GFP (control vector), pUAST-Hiw, or Hiw-ΔRING. All cells were co-transfected with pMT-Gal4 and induced with 0.5 mM copper sulfate for 24 h. Hiw is not expressed endogenously in S2R+ cells, and numerous breakdown products are observed for the ectopically expressed Hiw protein. The reduction in ectopic HA-Nmnat levels in lane 3 indicates that Hiw is capable of post-transcriptionally regulating Nmnat, and that the RING domain is required for this activity (lane 4). (G) Nmnat and Hiw-ΔRING form a physical interaction. Co-immunoprecipitation assays were performed from S2R+ cells lysate either co-transfected with pUAST-HA::Nmnat and pUAST-Hiw-ΔRING (lane 5) or mixed lysates from individual pUAST-HA::Nmnat and pUAST-Hiw-ΔRING transfections (lane 6). HA-Nmnat was immunoprecipitated by mouse anti-HA antibody against the HA tag on Nmnat. Despite the fact that Hiw-ΔRING was significantly degraded in S2R+ cell lysate (detected by Western blotting for Hiw antibody), a significant portion of Hiw-ΔRING protein co-immunoprecipitated with HA-Nmnat. The Input lanes (1–3) represent 1/25 of the total extract used for each immunoprecipitation. Scale bars = 12.5 µm; error bars represent standard error; ***p<0.001 in *t*-test.

The *hiw* mutation led to an increase in the levels of transgenic Nmnat, which was expressed via the ectopic Gal4/UAS promoter. We confirmed that the *hiw* mutation did not increase expression from the different Gal4 drivers used (ppk-Gal4, OK6-Gal4, and BG380Gal4, unpublished data). Hence the regulation of Nmnat by Hiw takes place post-transcriptionally. To test whether Nmnat is regulated by ubiquitination, we overexpressed the yeast ubiquitin protease *UBP2* in neurons, which can counteract the function of ubiquitin ligases [34],[42]. We found that co-expression of *UBP2* in neurons with the *HA-nmnat* transgene caused an increase in the levels of HA-Nmnat protein (Figure 7A and 7C), resembling the *hiw* mutant (Figure 6). This suggests that the levels of *Drosophila* Nmnat are controlled by ubiquitination.

We next tested whether the action of the Hiw E3 ubiquitin ligase is sufficient to modify Nmnat protein level in axons and synapses. Co-overexpression of *hiw* cDNA (*O/E hiw*) with *HA-nmnat* caused a strong decrease in HA-Nmnat protein in motoneuron axons (Figure 7B and 7C). Because Nmnat protein was difficult to detect at the NMJ (Figure 6F), we also examined the nerve terminals of class IV sensory neurons, whose concentrated location in the ventral nerve cord was easier to visualize. *O/E hiw* caused a reduction in HA-Nmnat protein in sensory axon terminals (Figure 7D and 7E). In contrast, co-expression of the dominant negative *hiw*-Δ*RING* mutation caused an increased level of HA-Nmnat in the sensory axon terminals (Figure 7D and 7E). Further evidence that Hiw function is sufficient to down-regulate Nmnat comes from studies in S2R+ cells, which do not express Hiw endogenously. Co-expression of Hiw, but not of Hiw-ΔRING, led to down-regulation of HA-Nmnat protein (Figures 7F and S4A). These findings suggest that Hiw plays a direct role in regulating the levels of Nmnat protein.

Curiously, we were unable to obtain evidence that Hiw down-regulates Nmnat via the UPS. Inhibition of the proteasome by addition of MG132, using several different concentrations and periods of time that affect known targets to the UPS (Materials and Methods) [43],[44], did not affect the down-regulation of Nmnat by Hiw in S2R+ cells (Figure S4A). To inhibit the proteasome in vivo we co-expressed dominant-negative proteasome subunit mutations, *DTS5* and *DTS7*, which in previous studies had been shown to lead to allow targets of the UPS to accumulate [45]–[47]. This led to only minor (7%) changes in the levels of HA-Nmnat in sensory neuron terminals (Figure S4B). Surprisingly, inhibition of the proteasome had a much greater effect upon HA-Nmnat levels in a *hiw* null mutant than in a wild-type background (Figure S4C). This observation does not favor a simple model that Hiw regulates Nmnat via the UPS. Instead, the data suggest that additional ubiquitin ligases may regulate Nmnat, and that the regulation of Nmnat may be more sensitive to the UPS when *hiw* is absent.

While the above data indicate that ubiquitination is important for the regulation of Nmnat, the detailed mechanism by which Hiw regulates Nmnat remains to be determined. The mechanism may involve a direct interaction, since co-immunoprecipitation experiments indicate that Nmnat can robustly interact with the enzyme dead Hiw-ΔRING protein in S2R+ cells (Figure 7G).

### Highwire Promotes Destruction of Nmnat in the Distal Stump of Injured Axons

**Figure 8:**
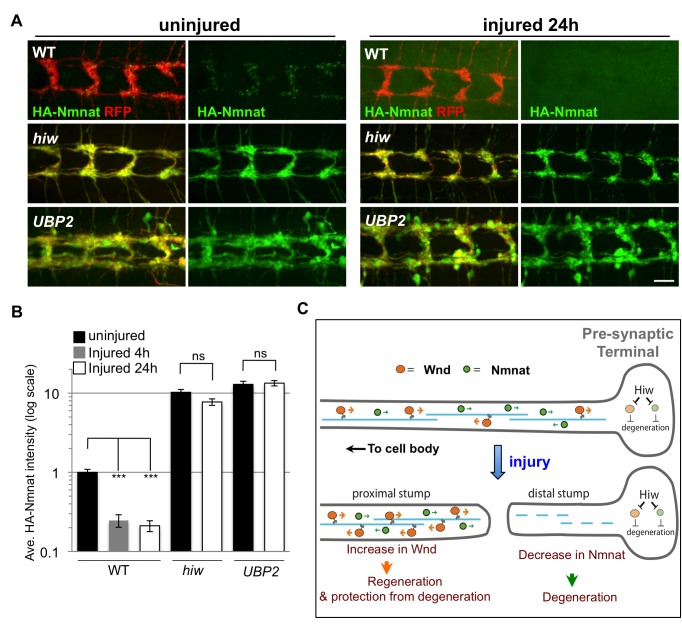
Hiw promotes Nmnat protein turnover in the injured distal axons and synapses (A) Distal axons and synapses of *ppk-Gal4,UAS-mCD8::RFP* labeled sensory neurons, located in the ventral nerve cord, either before or 24 h after injury. Transgenic *HA::nmnat* is expressed in a wild-type (WT) genetic background *(UAS-HA::nmnat/+; ppk-Gal4,UAS-mCD8::RFP/+)*, *hiw* ^Δ*N*^ mutant *(hiw* ^Δ*N*^ *; UAS-HA::nmnat/+; ppk-Gal4,UAS-mCD8::RFP/+)* or co-expressed with *UBP2* (*UAS-HA::nmnat/UAS-UBP2; ppk-Gal4,UAS-mCD8::RFP/+*). Consistent with observations in motoneurons (Figure 6), mutation of *hiw* or co-expression of *UBP2* causes a dramatic elevation in HA-Nmnat protein, and this does not disappear after injury, in dramatic contrast to the disappearance of HA-Nmnat in wild-type (WT) animals. Of note, the nerve terminals of the *ppk-Gal4* labeled axons appear to be overgrown in *hiw* and *UBP2* expressing mutants, similar to previous descriptions in other neuron types [8],[41]. Hence the quantification shown in (B) is normalized to the size of the nerve terminals (labeled by mCD8-RFP). Due to the expression of *HA-nmnat*, no axons are degenerating in any of the above genotypes. (B) Quantification of the average HA-Nmnat intensity in *ppk-Gal4* expressing sensory neuron axon terminals, within the most posterior four segments of the ventral nerve cord. Error bars represent standard error. ns, not significant, *p*>0.05. (C) Model: Hiw promotes multiple independent responses to injury, through independent pathways. Hiw promotes degeneration in the distal stump by down-regulating Nmnat, and concurrently regulates regeneration (and protection from degeneration [15],[30]) in the proximal stump by regulating Wnd. Since Hiw may localize and function in distal axons, injury may relieve the inhibition of Wnd by Hiw in the proximal stump. Scale bars = 12.5 µm, error bars represent standard error; ***p<0.001; ns, not significant, *p*>0.05 in *t*-test.

A recent study using vertebrate cultured neurons suggested that the disappearance of Nmnat2, which has a short half-life, from the distal stump of axons may serve as a trigger for the Wallerian degeneration process [21]. This leads to an attractive hypothesis that Hiw promotes the disappearance of Nmnat protein from the distal stump. Supporting this model, we observed that HA-Nmnat levels become significantly reduced in axons (Figure S5A) and synapses (Figure 8) distal to the injury site. In contrast, HA-Nmnat levels increase in the proximal stump after injury (Figure S5A), consistent with the model that a cytoplasmic form of this enzyme is transported in axons from the cell body [21]. Within 4 h after injury, the majority of HA-Nmnat in sensory axon terminals had disappeared (Figure 8). By comparison, a significant amount of green fluorescent protein (GFP)-Hiw remained at this time point (Figure S5B).

When *hiw* was mutant, the levels of HA-Nmnat in the distal stump did not decrease significantly below its starting level, even 24 h after injury (Figure 8A and 8B). Expression of *UBP2* had a similar effect upon HA-Nmnat in the distal stump after injury (Figure 8A and 8B). These findings indicate that Hiw and the ubiquitination are required for the disappearance of Nmnat protein in the distal stump.

### Highwire Can Specifically Down-regulate Mouse Nmnat2 Protein in *Drosophila* Neurons

**Figure 9:**
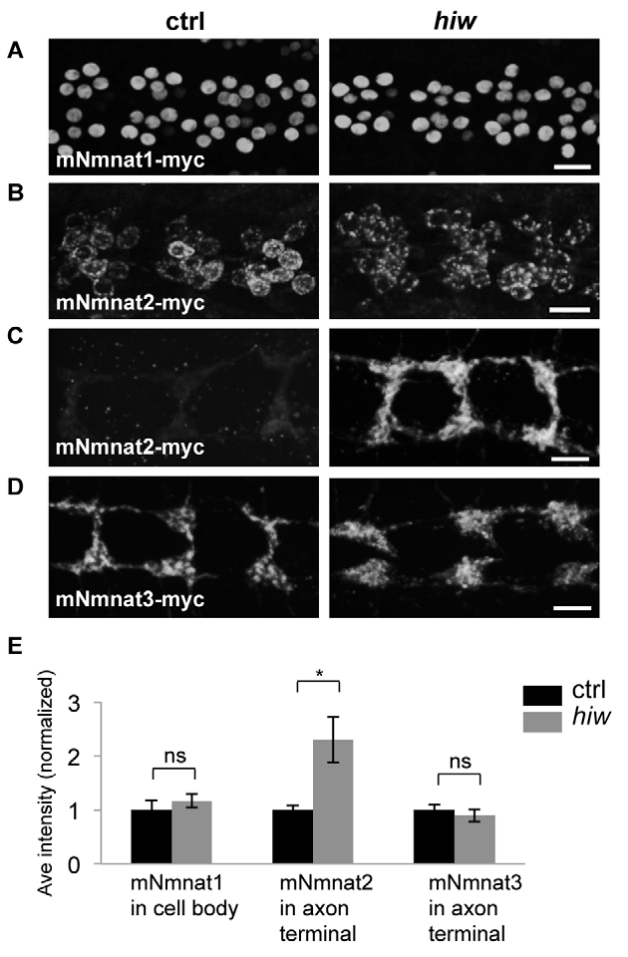
Hiw is capable of regulating mouse Nmnat2, but not mouse Nmnat1 or Nmnat3, protein. (A, B) UAS-mNmnat1::myc and UAS-mNmnat2::myc transgenes, expressed in motoneurons with the BG380-Gal4 driver, show no difference in cell body levels between control and *hiw^ND8^* mutants. (C–D) UAS-mNmnat2::myc, and UAS-mNmnat3::myc were expressed with ppk-Gal4 in order to visualize localization at sensory neuron axon terminal in the nerve cord. The levels of mNmnat2-myc protein at axon terminals were increased in *hiw^ND8^* mutants. In contrast, mNmant3-myc protein levels were similar in between control and *hiw^ND8^* mutant genotypes. (E) Quantification of average intensity of mNmnat1 in cell body, and mNmnat2 and mNmnat3 in axon terminals from (A–D). Scale bars = 12.5 µm, error bars represent standard error; *p<0.05; ns, not significant, *p*>0.05 in *t*-test.

Vertebrates utilize three distinct Nmnat enzymes, which localize to distinct subcellular locations. We tested whether Hiw was capable of influencing the levels of ectopically expressed mouse Nmnat1, which localizes to nuclei, mouse Nmnat2, which co-localizes with golgi and late endosome markers, or mouse Nmnat3, which localizes to mitochondria [48]–[50], by crossing UAS-mNmnat1::myc, UAS-mNmnat2::myc, and UAS-mNmnat3::myc transgenes [51],[52] into the *hiw* mutant background. Intriguingly, mutations in *hiw* resulted in increased levels of mNmant2-myc protein within axons and synaptic terminals of class IV sensory neurons (Figure 9). This finding implies that mNmant2-myc protein can be transported to distal axons and synapses, and that mouse Nmnat2 shares a conserved protein feature with *Drosophila* Nmnat that allows it to be regulated by Hiw. In contrast, loss of *hiw* had no effect upon the levels of mNmnat1 or mNmnat3. We interpret that the distinct subcellular localization of mNmnat2 may make this protein more susceptible to regulation by Hiw, and that that a conserved mechanism, involving Hiw homologues, may regulate Nmnat2 in vertebrate neurons.

## Discussion

### Highwire Promotes Degeneration by Down-regulating Nmnat Protein

Since the discovery of the dramatic inhibition of degeneration by the *WldS* mutation, many studies have focused upon the action of the NAD+ biosynthetic enzyme isoforms, Nmnat1, Nmnat2, and Nmnat3, which in some circumstances can confer protection against axonal degeneration (reviewed in [22],[23]). Most of these studies involve gain-of-function overexpression experiments; it has been difficult to address the role of endogenous Nmnat enzymes in this process. Recent observations indicate that endogenous Nmnat activity plays an essential role in neuronal survival, and its depletion leads to neurodegeneration [21],[25]–[27]. In addition, recent studies in vertebrate neurons suggest that the cytoplasmic isoform, Nmnat2, has a short half-life in neurons [21]. An attractive model proposes that Nmnat2 is rapidly turned over in axons, and that its loss in the distal stump of an axon, which has become disconnected from its cell body, leads to the initiation of Wallerian degeneration [21].

Some aspects of this model are supported by our current in vivo characterization in *Drosophila*. We have identified Hiw, a highly conserved protein with features of an E3 ubiquitin ligase, as an important regulator of Wallerian degeneration. Hiw's role in this process involves the Nmnat protein, whose levels in axons and synapses are regulated post-transcriptionally by Hiw function. In *hiw* mutants, Wallerian degeneration is strongly inhibited, and the increased level of Nmnat protein in *hiw* mutants is both required and sufficient to inhibit degeneration.

While the localization of endogenous Hiw in *Drosophila* is not known, homologues in mice and *Caenorhabditis elegans* have been detected in axons and at synapses [9],[53], so it is in the appropriate location to target the destruction of Nmnat in distal axons (Figure 8C). However, it remains to be determined whether the down-regulation of Nmnat in the distal stump per se is the trigger for Wallerian degeneration. When HA-Nmnat was overexpressed, axons were protected from degeneration long after the rapid disappearance of detectable protein in the distal stump. It is possible that even very low levels of Nmnat protein are sufficient to protect from degeneration. It is also formally possible that the basal levels of Nmnat before injury, rather than the disappearance of Nmnat after injury, is an important determinant of degeneration. We also acknowledge that axonal degeneration likely involves additional steps downstream or in parallel to the regulation of Nmnat by Hiw. While overexpression of Hiw can induce a reduction in HA-Nmnat levels (Figure 7), we were unable to observe an enhanced rate of degeneration when Hiw was overexpressed.

### The Relationship of Highwire and the UPS

Studies almost a decade ago suggested a role for the UPS in the initiation of Wallerian degeneration [3]. It is tempting to propose that this role is manifested by the regulation of Nmnat by Hiw. However our observations caution against a simple interpretation that Hiw regulates Nmnat via the UPS, since Hiw can promote disappearance of Nmnat protein in cells in a manner unaffected by proteasome inhibitors (Figure S4A). Moreover, in vivo, inhibition of the proteasome had only a minor effect upon Nmnat levels in a wild-type background (Figure S4B and S4C). However in *hiw* mutants, Nmnat levels were very sensitive to the function of the proteasome (Figure S4C). We interpret that additional ubiquitin ligases and the UPS may regulate Nmnat independently of Hiw.

Regardless of the role of the proteasome, our observations suggest that ubiquitin plays an important role in Nmnat regulation. Overexpression of the yeast de-ubiquitinating protease *UBP2* leads to increased levels of Nmnat protein and inhibition of Wallerian degeneration, in a manner that requires endogenous Nmnat (Figure S3). Future studies of the mechanism by which Hiw regulates Nmnat will therefore consider potential proteasome-independent roles of ubiquitination. Of note, in yeast UBP2 has been shown to preferentially disassemble polyubiquitin chains linked at Lys63 [54], which have been found to perform non-proteolytic functions in DNA repair pathways [55], kinase activation [56], and receptor endocytosis [57],[58]. We should also consider the possibility that Hiw regulates Nmnat indirectly: since we have thus far been unable to detect any ubiquitinated Nmnat species, it is possible that an intermediate, yet unknown, regulator of Nmnat may be the actual substrate of ubiquitination. Nevertheless, co-immunoprecipitation studies from S2R+ cells indicate that Hiw and Nmnat have the capacity to interact (Figure 7G).

### Highwire Can Regulate Mouse Nmnat2

The mechanism and cellular location of Nmnat's protective action is a highly debated subject. Observations in the literature point to both NAD+-dependent and NAD+-independent models for the strong protection by the *WldS* mutation [23]. The location of its protective action may be the mitochondria, since mitochondrially localized Nmnat can protect axons from degeneration [51],[52],[59]. However golgi/endosomal localized Nmnat2 can also be protective [21],[27],[60],[61]. Our findings suggest that mutation of *hiw* leads to an increase in the pool of endogenous Nmnat that functionally impacts degeneration.

While the site of endogenous Nmnat function during axonal degeneration remains to be identified, we found that the levels of ectopically expressed mouse Nmnat2 were specifically increased in the *hiw* mutant background. In contrast, the levels of nuclearly localized mNmnat1 or mitochondrially localized mNmnat3 were unaffected by Hiw. Since Nmnat2 has a short half-life in vertebrate neurons [21], it is intriguing to propose that it is regulated by Hiw orthologs via an analogous mechanism.

Since Nmnat2 does not appear to localize to mitochondria, does this favor a non-mitochondrial activity, such as function as a chaperone [62],[63], for the protective action? It remains challenging to determine the exact location of protection, since the most apparent changes in Nmnat protein may not necessarily be the functionally relevant changes.

### Multiple Roles of Highwire in Responses to Injury

A previously characterized target of Hiw regulation is the Wnd MAP kinase kinase kinase [16],[17]. This axonal kinase is also capable of inhibiting Wallerian degeneration in motoneurons [20]. The protective action of Wnd requires a downstream signaling cascade and changes in gene expression mediated by the Fos transcription factor [20]. Loss of *nmnat* does not affect this signaling cascade (unpublished data) nor does it affect the protective action of Wnd (Figure 5C and 5D). Conversely, loss of *wnd* does not affect the protection caused by overexpressing *nmnat* (Figure 5A and 5B). Importantly, the regulation of Nmnat by Hiw does not appear to require Wnd function, and Wnd and Nmnat can protect axons independently of each other. These findings favor the model that Wnd and Nmnat are both regulated by Hiw and influence axonal degeneration through independent mechanisms.

The Wnd kinase plays additional roles in neurons, which can be genetically separated from Nmnat function. These include regulation of synaptic growth: a dramatic synaptic overgrowth phenotype in *hiw* mutants is fully suppressed by mutation of *wnd*, but is not at all affected by knockdown of *nmnat* (Figure 4E). Wnd/DLK also promotes axonal sprouting in response to axonal injury [30], which is also unaffected by *nmnat* knockdown (unpublished data). It is therefore clear that by regulating both Wnd and Nmnat, Hiw regulates multiple independent pathways in neurons.

It is intriguing that the actions of both Wnd and Nmnat promote cellular responses to axonal injury. Axonal regeneration requires an initiation of a growth program within the axon, which depends upon the function of Wnd and its homologues [28]–[32]. Equally important is a clearance of the distal stump to make room for the regenerating axon [64]–[66]. Since both Wnd and Nmnat are transported in axons [21],[30], Figure 8C proposes a model in which Hiw function in the distal axon terminal could simultaneously promote destruction of Nmnat in the distal stump, and accumulation of Wnd in the proximal stump. The latter is observed after injury [30], and is required to promote new axonal growth. The actual location in which Hiw regulates Nmnat remains to be determined. As an upstream regulator of both sprouting in the proximal stump and degeneration of the distal stump, Hiw may play a central role in regulating the ability of a neuron to regenerate its connection after injury.

Importantly, the protective action of Nmnat may not be limited to Wallerian degeneration. The *WldS* mutation can protect neurons from degeneration in a wide variety of paradigms, from models of neurodegenerative disease, diabetic neuropathy, excitotoxity, and loss of myelination [22],[23]. These findings suggest that action and regulation of Nmnat function is broadly important for neuronal function and maintenance. As a critical regulator of Nmnat, the Hiw ubiquitin ligase and its vertebrate homologues deserve further scrutiny for potential roles in human health and disease.

## Materials and Methods

### Fly Stocks

The following strains were used in this study: *Canton-S* (wild-type), *hiw^ND8^* [8], *hiw* ^Δ*N*^, *UAS-hiw* and *UAS-hiw*-Δ*RING* from [67], *OK6-Gal4* [68], *BG380-Gal4* [69] *m12-Gal4* (P(GAL4)^5053A^) [70], *ppk-Gal4* [71], *Or47b-Gal4* [72], *UAS-UBP2* [41], *UAS-DTS5*, and *UAS-DTS7* from [45], *wnd^1^*, *wnd^3^*, and *UAS-wnd* from [16]. *UAS-HA::nmnat* [25], *UAS-WldS* [2], *UAS-mNmnat1::myc*, *UAS-mNmnat2::myc*, and *UAS-mNmnat3::myc* [51],[52], and *UAS-Dcr2* were gifts from Grace Zhai, Liqun Luo, Marc Freeman, and Stephan Thor. *UAS-wnd-RNAi* (Construct ID 13786) and *UAS-nmnat-RNAi* (construct ID 32255) were acquired from the Vienna RNAi center [37].

### Nerve Crush Assay

The segmental nerves of third instar larvae were visualized through the cuticle under a standard dissection stereomicroscope. While larvae were anesthetized with CO_2_ gas, the segmental nerves were pinched tightly through the cuticle for 5 s with Dumostar number 5 forceps. After successful injury, the posterior half of the larva was paralyzed. Larvae were then transferred to a grape plate and kept alive for varying periods of time at 25°C. Also see [30].

### Immunocytochemistry

Larvae were dissected in PBS and fixed in 4% paraformaldehyde or formaldehyde in PBS for 25 min for the following antibodies used: ms anti-Futsch (1∶100), guinea pig (gp) anti-NMNAT [25], (gift from Hugo Bellen and Grace Zhai, 1∶1,000), rat anti-HA (Roche, 1∶100), rat anti-elav (1∶50), or fixed in Bouin's fixative for 15 min for the following antibodies: ms anti-Brp (1∶200), Rb anti-GluRIII (1∶1,000 [73]), Rb anti-DVLGUT (1∶10,000, [74]). Rat anti-elav (7E8A10) and ms anti-Brp (NC82) were obtained from Developmental Studies Hybridoma Bank, University of Iowa. The conjugated secondary antibodies are used and diluted as follows: Cy3-Gt anti-HRP and Cy5-Gt anti-HRP (from Jackson labs) at 1∶200, A488-Rb anti-GFP (from Molecular Probes) at 1∶1,000. For secondary antibodies Cy3 and Alexa-488 conjugated Goat anti-rb/mouse/rat/gp (from Invitrogen) were used at 1∶1,000. All antibodies were diluted in PBS-0.3%Triton with 5% normal goat serum.

### Imaging

Confocal images were collected at room temperature on an Improvision spinning disk confocal system, consisting of a Yokagawa Nipkow CSU10 scanner, and a Hamamatsu C9100-50 EMCCD camera, mounted on a Zeiss Axio Observer with 25× (0.8 NA) multi and 40× (1.3NA), 63× (1.5NA), and 100× (1.46 NA) oil objectives. Similar settings were used to collect all compared genotypes and conditions. Volocity software (Perkin Elmer) was used for all measurements of average and total intensities.

For measurement of Nmnat intensity in the neuropil, the neuropil area was selected based on co-staining for the synaptic marker Brp. Objects meeting intensity criteria of >0.8 standard deviations above the mean were selected within a 140-µm long region of the ventral nerve cord and then summed for total intensity. The average intensity of the HA-Nmnat staining in muscle 4 NMJs was measured within the synaptic area defined by HRP staining after subtraction of background intensity for each image. The average intensity of the HA-Nmnat staining in motoneuron axons and sensory nerve terminus was measured with a similar protocol. Likewise for neuronal nuclei, the average intensity for Nmnat staining was measured in neuronal nuclei defined by staining for the neuronal marker Elav. Numbers are shown normalized to the average intensity of the control for each figure.

### Quantification of Degeneration

To quantify axonal degeneration, we scored (while blind to genotype) the fragmentation of *m12-Gal4*, *UAS-mCD8-GFP* labeled axons within segmental nerves according to one of five categories between 0 and 100% (with 100% meaning completely degenerated) as described in [20]. All measurements indicate the average from >100 axons.

To quantify the degeneration of the NMJ, NMJs were stained for the MAP1B homologue Futsch and axonal membrane marker HRP, and were scored into one of three categories: (1) complete degeneration, defined by a complete loss of Futsch staining from the NMJ and fragmentation of the axonal membrane, (2) partial degeneration, defined by a partial loss of Futsch staining from the NMJ and partial membrane fragmentation, and (3) no degeneration, in which there was no fragmentation of the membrane or Futsch, similar to uninjured control animals. All quantifications shown represent the average scores from multiple NMJs from >six animals quantified in duplicate by two independent observers who were blind to the genotype.

Degeneration of ORN axons was quantified following the previously defined method [2],[35] by calculating the percentage of brains for each genotype in which contralateral axon projections could still be detected.

For all the statistical analysis, Student's *t* test was applied.

### Electrophysiology

Intracellular recordings were made from muscle 6 in segments A3 and A4 of third-instar male larvae. The larvae were visualized with oblique illumination on an Olympus BX51W1 fixed stage upright microscope with a 10× water immersion objective. Sharp electrodes (15–25 MΩ), made of borosilicate glass (outer diameter 1.2 mm) were filled with 3 M KCl. The signal was amplified with a Geneclamp 500B (Molecular Devices), digitized with a Digidata 1322A interface (Molecular Devices), and stored on a PC with pClamp 10.2 (Molecular Devices). Recordings were performed in HL3 Stewart saline [75] that contained (in mM) 70 NaCl, 5 KCl, 20 MgCl_2_, 10 HCO_3_, 5 trehalose, 115 sucrose, 5 HEPES, 1 CaCl_2_,, the pH was adjusted to 7.2. For all genotypes the resting membrane potentials and input resistances were similar, with average resting potentials of −73±4 and input resistances of 6±0.2 MΩ. To elicit evoked EJPs, the nerve was drawn into a tight-fitting suction electrode and stimulated with brief (1 ms) depolarizing pulses controlled with Digidata interface. The stimulus amplitude was set to 125% of the amplitude necessary to activate the higher threshold of the two excitatory axons that innervate the muscle. For injured wild-type larvae (in which nerve stimulation did not produce evoked synaptic responses) the stimulus amplitude was set to double the amplitude used for un-injured larvae. However evoked responses were not observed, even at the largest stimulus amplitude that the equipment could produce. For analysis of evoked responses, 100 events per cell recorded at 0.2 Hz were measured using the cursor feature in Clampfit 10.2 (Molecular Devices) and then averaged. For analysis of spontaneous miniature EJPs, at least 50 consecutive events were measured per cell using MiniAnal (Synaptosoft). mEJP frequency was calculated from the first 30 s of recording time.

### Cell Culture

S2R+ cells were cultured in Schneider's medium (Gibco) which contains 10% (v/v) FBS (Gibco) and 1% penicillin-streptomycin (Invitrogen). For plasmid transfection, cells were transfected using FuGENE 6 (Promega) following the manufacturer's instructions. Copper sulfate solution (0.5 mM) was added 6 h after transfection to induce plasmid expression. Cell lysates were collected after 24 h. Plasmids used for transfection were pMT-Gal4 [76], pUAST-eGFP [77], pUAST-GFP-Hiw [67], pUAST-HiwΔRING [67], and pUAST-HA-Nmnat [25].

To inhibit the UPS, cells were treated with MG132 (InSolution, Calbiochem) or DMSO as control using several different conditions: 25 µM for 6 h, 5 µM for 20 h, and 5 µm for 36 h. All of these conditions led to an increase in the levels of polyubiquitinated proteins, detected by Western blots probed with FK1 antibodies.

### Biochemistry

The following antibodies were used for Western blotting: rb anti-Hiw (ref, 1∶2,000), rat anti-HA (Flourochem, 1∶2,500), ms anti-β-tubulin (1E7) and ms anti-β-catenin (armadillo, N27A1) from Developmental Studies Hybridoma bank (University of Iowa), ms anti-polyubiquitin, (FK1, Enzo Life Sciences, 1∶1,000), and ms anti-ubiquitin (P4D1, Cell Signaling, 1∶1,000). Westerns were probed with IRDye 800CW and 680RD conjugated secondary antibodies (LiCor biosciences, 1∶10,000) and imaged for quantitative analysis via a LiCor Odyssey imaging system.

S2R+ cells were transfected with either pUAST-HiwΔRING or pUAST-HiwΔRING and pUAST-HA-Nmnat. Cells from 6-cm dishes were harvested in 500-µl ice-cold lysis buffer (20 mM HEPES [pH 7.5]), 200 mM KCl, 0.05% Triton X-100, 2.5 mM EDTA, 5 mM DTT, 5% glycerol and Complete proteinase inhibitor [Promega]). 1.5 mg Protein G conjugated Dynabeads (Invitrogen) were used to capture 10 µl mouse monoclonal anti-HA antibody (HA-7 ascites fluid, Sigma) at room temperature for 30 min, and were then incubated with cell lysates for 30 min at room temperature. The immunoprecipitates were then washed three times with ice-cold lysis buffer and subjected to Western blotting analysis.

## Supporting Information

Figure S1 **Synaptic markers remain intact in** ***hiw*** **mutants after injury.** Representative muscle 4 NMJs for WT (*Canton S*) or *hiw (hiw^ΔN^)* mutants stained in (A) for Futsch (green), Dvglut (synaptic vesicles, red), and HRP (neuronal membrane, blue). In (B) NMJs are stained for GluRIII (post-synaptic GluR receptor subunit [49], green), Brp (pre-synaptic active zones, red), and HRP (axonal membrane, blue), before or 24 h after injury. While *hiw* mutants have reduced Dvglut staining ([12] and A) and smaller synaptic Brp and GluRIII puncta (B), there is no noticeable difference between the uninjured and injured NMJs. Scale bars = 12.5 µm.
(TIF)

Figure S2 **Endogenous Nmnat in** ***Drosophila*** **motoneurons.** Depletion of Nmnat in larval motoneurons by expression of *nmnat-RNAi*. Expression of UAS-*nmna*t-*RNAi* with a pan-neuronal Gal4 driver (*BG380-Gal4, UAS-Dcr2; UAS-nmnat-RNAi/+)* depletes Nmnat staining (green) in neuronal nuclei (marked by co-staining with Elav, red) but not in neighboring glial cells (for which Nmnat staining increased). Quantification of the reduced staining in neuronal nuclei suggested that the Nmnat levels were reduced to 49.3% of wild-type levels in motoneurons (*p*<0.01, *n* = 6.7). (A) Depletion of *nmnat* by expression of *nmnat-RNAi* in neurons does not affect NMJ morphology. Representative muscle 4 NMJs stained for Futsch (green), Dvglut (synaptic vesicles, red), and HRP (neuronal membrane, blue). We did not observe spontaneous axonal or synaptic degeneration when *nmnat* was depleted by RNAi, probably because the depletion was not complete. (B) In *hiw (hiw* ^Δ*N*^ *)* mutants, endogenous Nmnat (green) is reduced in neuronal nuclei. Similarly to the *nmnat RNAi* knockdown in (A), Nmnat staining increases in neighboring glial cells. (C) Injury signaling via Wnd may down-regulate nuclear Nmnat. Nmnat protein disappears from neuronal nuclei and appears in neighboring glial cells 24 h after injury. A similar change occurs in *hiw* mutants (C), and when Wnd is overexpressed in neurons *(BG380-Gal4; UAS-wnd/+)*. Conversely, *wnd* loss-of-function mutants *(wnd-1/wnd-2)* have increased levels of nuclear Nmnat. Because Wnd becomes activated by axonal injury, we expect that these changes in nuclear Nmnat are mediated by a common mechanism. The functional relevance of these changes is not yet clear. (D) Quantification of average nuclear Nmnat intensity, normalized for wild type, for experiments in (D). Scale bars = 12.5 µm.
(TIF)

Figure S3 **Inhibition of degeneration by UBP2 requires Nmnat function.** (A) *m12-Gal4*, UAS-*mCD8::GFP* labeled single axons (green) 24 h after injury in animals co-expressing *UBP2* with a control UAS line *(UAS-Dcr2; UAS-UBP2/+; UAS-nls::DsRed-(Ctrl)/m12-Gal4, UAS-mCD8::GFP)* or co-expressing *UBP2* when with UAS-*nmnat RNAi* to reduce endogenous Nmnat *(UAS-Dcr2; UAS-UBP2/UAS-nmnatRNAi; m12-Gal4, UAS-mCD8::GFP/+)*. (B) Degeneration index for the *m12-Gal4, UAS-mCD8::GFP* labeled axons for genotypes in (A). Scale bars = 12.5 µm; error bars represent standard error; ***p<0.001 in *t*-test.
(TIF)

Figure S4 **Hiw down-regulates Nmnat independently of the UPS.** (A) The decrease in Nmnat levels promoted by Hiw induction in S2R+ cells was not diminished when the proteasome was inhibited. S2R+ cells co-transfected with pUAST-HA::Nmnat and pUAST-GFP or pUAST-GFP::Hiw, then were incubated with DMSO vehicle or 5 µM MG132 for 20 h. The levels of HA-Nmnat are compared by Western blotting for the HA epitope. Relative levels compared to the β-tubulin standard were measured on the LiCor Odyssey system. Similar results were observed for additional concentrations and time points (25 µM MG132 for 6 h, and 5 µM MG132 for 12 h, unpublished data). (B) Inhibition of the proteasome in sensory neurons only modestly changes HA-Nmnat levels. Average HA-Nmnat intensity in ppk sensory neuron axon terminals was compared between wild-type (*UAS-HA::nmnat/+; ppk-Gal4,UAS-mCD8::RFP/+*), and animals co-expressing *DTS5* and *DTS7* to inhibit the proteasome *(UAS-HA::nmnat/UAS-DTS5; ppk-Gal4,UAS-mCD8::RFP/UAS-DTS7)*. Data are shown for two conditions: flies raised continuously at 25°C, and flies raised at 25°C, then shifted to 30°C for 2 d. Error bars represent standard error. *p<0.05. (C) Hiw and the UPS may influence Nmnat levels cooperatively. Total protein from third instar larval brains or young adult heads processed for Western blot from animals co-expressing *DTS5* and *DTS7* [45] to inhibit the proteasome (raised at 25°C), and compared to animals co-expressing two control UAS-*PKC* transgenes. Relative levels of HA-Nmnat protein, compared to the β-catenin standard, were measured on the LiCor Odyssey system. Combination of the *hiw* mutation with inhibition of the proteasome leads to much higher levels of HA-Nmnat, suggesting that Hiw and the UPS may potentially influence HA-Nmnat independently, rather than through the same pathway. Error bars represent standard error; *p<0.05 in *t*-test.
(TIF)

Figure S5 **Changes in HA-Nmnat protein level in proximal and distal stumps, contrasted with GFP-Hiw after injury.** (A) Quantification of average HA-Nmnat intensity in motoneurons axons for wild-type *(OK6-Gal4/UAS-HA::nmnat) or hiw* mutants (*hiw^ΔN^*;*OK6-Gal4/UAS-HA::nmnat)* before (black) or 8 h after (gray) injury. HA-Nmnat levels in both proximal and distal axons were quantified and normalized to the average HA-Nmnat intensity in uninjured WT animals as described in Materials and Methods. Injury induces an increase of HA-Nmnat in the proximal stump in both wild-type and *hiw* mutant backgrounds. However, in the distal stump, the levels of HA-Nmnat reduced by 60.7% within 8 h in WT animals, but remained constant in *hiw* mutants. (B) Quantification of average HA-Nmnat and GFP-Hiw intensity in axon terminals of *ppk-Gal4,UAS-mCD8::RFP* labeled sensory neurons before or 4 h after injury. Error bars represent standard error; **p<0.01; ***p<0.001; ns, not significant, *p*>0.05 in *t*-test.
(TIF)

## Abbreviations

DLK: dileucine zipper kinase
EJP: excitatory junction potential
GFP: green fluorescent protein
Hiw: Highwire
NMJ: neuromuscular junction
Nmnat: nicotinamide mononucleotide adenyltransferase
*O/E*: *o*verexpression
UPS: ubiquitin proteasome system
Wnd: Wallenda
