# Supplementary figures and images for: The Highwire Ubiquitin Ligase Promotes Axonal Degeneration by Tuning Levels of Nmnat Protein
Source: PLoS Biol. 2012 Dec 4;10(12):e1001440. doi: 10.1371/journal.pbio.1001440 (PMC3514318; doi:10.1371/journal.pbio.1001440)

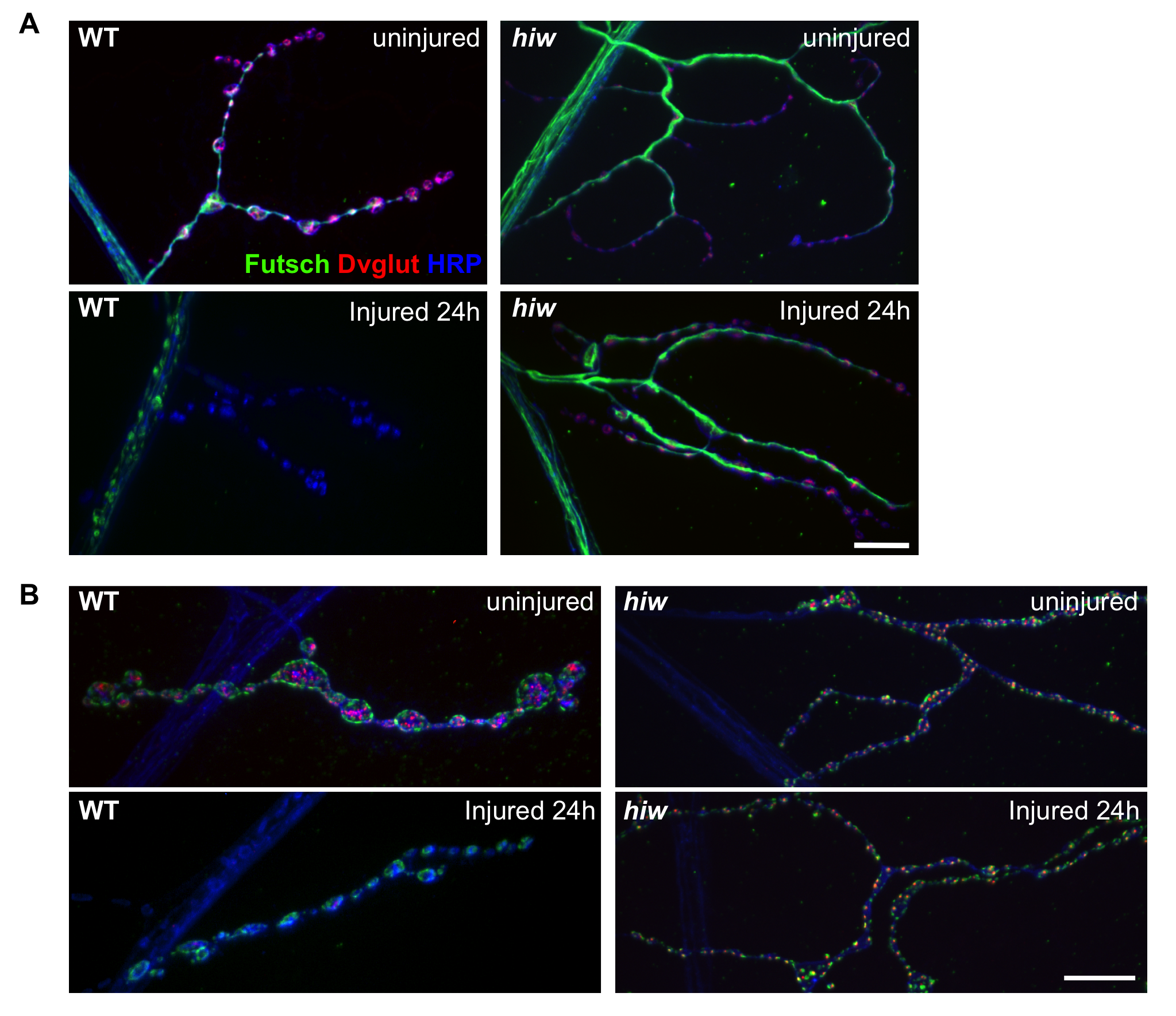

Supplement: Figure S1 — Synaptic markers remain intact in hiw mutants after injury. Representative muscle 4 NMJs for WT (Canton S) or hiw (hiwΔN) mutants stained in (A) for Futsch (green), Dvglut (synaptic vesicles, red), and HRP (neuronal membrane, blue). In (B) NMJs are stained for GluRIII (post-synaptic GluR receptor subunit [49], green), Brp (pre-synaptic active zones, red), and HRP (axonal membrane, blue), before or 24 h after injury. While hiw mutants have reduced Dvglut staining ([12] and A) and smaller synaptic Brp and GluRIII puncta (B), there is no noticeable difference between the uninjured and injured NMJs. Scale bars = 12.5 µm. (TIF) [file pbio.1001440.s001.tif]

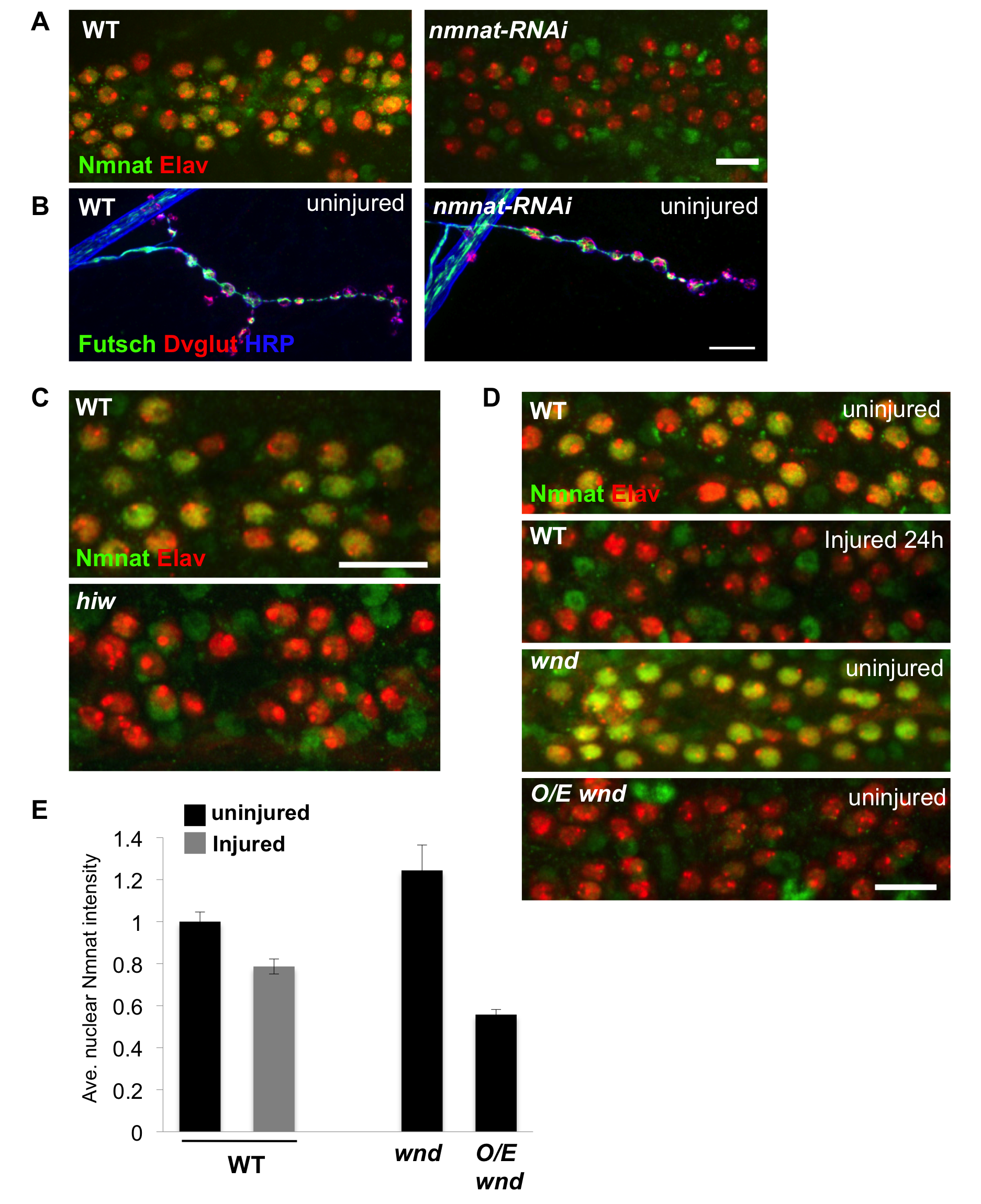

Supplement: Figure S2 — Endogenous Nmnat in Drosophila motoneurons. Depletion of Nmnat in larval motoneurons by expression of nmnat-RNAi. Expression of UAS-nmnat-RNAi with a pan-neuronal Gal4 driver (BG380-Gal4, UAS-Dcr2; UAS-nmnat-RNAi/+) depletes Nmnat staining (green) in neuronal nuclei (marked by co-staining with Elav, red) but not in neighboring glial cells (for which Nmnat staining increased). Quantification of the reduced staining in neuronal nuclei suggested that the Nmnat levels were reduced to 49.3% of wild-type levels in motoneurons (p<0.01, n = 6.7). (A) Depletion of nmnat by expression of nmnat-RNAi in neurons does not affect NMJ morphology. Representative muscle 4 NMJs stained for Futsch (green), Dvglut (synaptic vesicles, red), and HRP (neuronal membrane, blue). We did not observe spontaneous axonal or synaptic degeneration when nmnat was depleted by RNAi, probably because the depletion was not complete. (B) In hiw (hiw ΔN ) mutants, endogenous Nmnat (green) is reduced in neuronal nuclei. Similarly to the nmnat RNAi knockdown in (A), Nmnat staining increases in neighboring glial cells. (C) Injury signaling via Wnd may down-regulate nuclear Nmnat. Nmnat protein disappears from neuronal nuclei and appears in neighboring glial cells 24 h after injury. A similar change occurs in hiw mutants (C), and when Wnd is overexpressed in neurons (BG380-Gal4; UAS-wnd/+). Conversely, wnd loss-of-function mutants (wnd-1/wnd-2) have increased levels of nuclear Nmnat. Because Wnd becomes activated by axonal injury, we expect that these changes in nuclear Nmnat are mediated by a common mechanism. The functional relevance of these changes is not yet clear. (D) Quantification of average nuclear Nmnat intensity, normalized for wild type, for experiments in (D). Scale bars = 12.5 µm. (TIF) [file pbio.1001440.s002.tif]

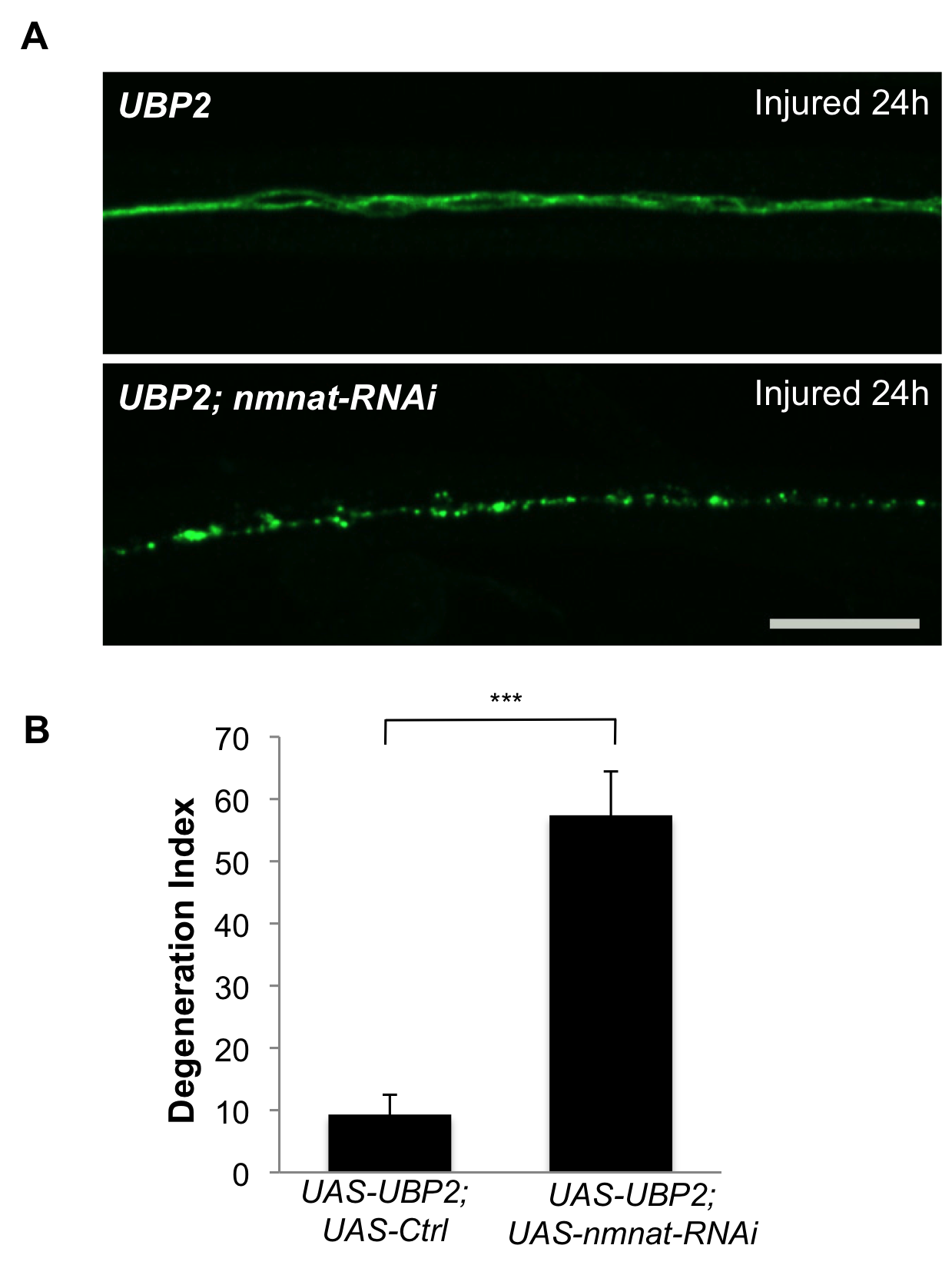

Supplement: Figure S3 — Inhibition of degeneration by UBP2 requires Nmnat function. (A) m12-Gal4, UAS-mCD8::GFP labeled single axons (green) 24 h after injury in animals co-expressing UBP2 with a control UAS line (UAS-Dcr2; UAS-UBP2/+; UAS-nls::DsRed-(Ctrl)/m12-Gal4, UAS-mCD8::GFP) or co-expressing UBP2 when with UAS-nmnat RNAi to reduce endogenous Nmnat (UAS-Dcr2; UAS-UBP2/UAS-nmnatRNAi; m12-Gal4, UAS-mCD8::GFP/+). (B) Degeneration index for the m12-Gal4, UAS-mCD8::GFP labeled axons for genotypes in (A). Scale bars = 12.5 µm; error bars represent standard error; ***p<0.001 in t-test. (TIF) [file pbio.1001440.s003.tif]

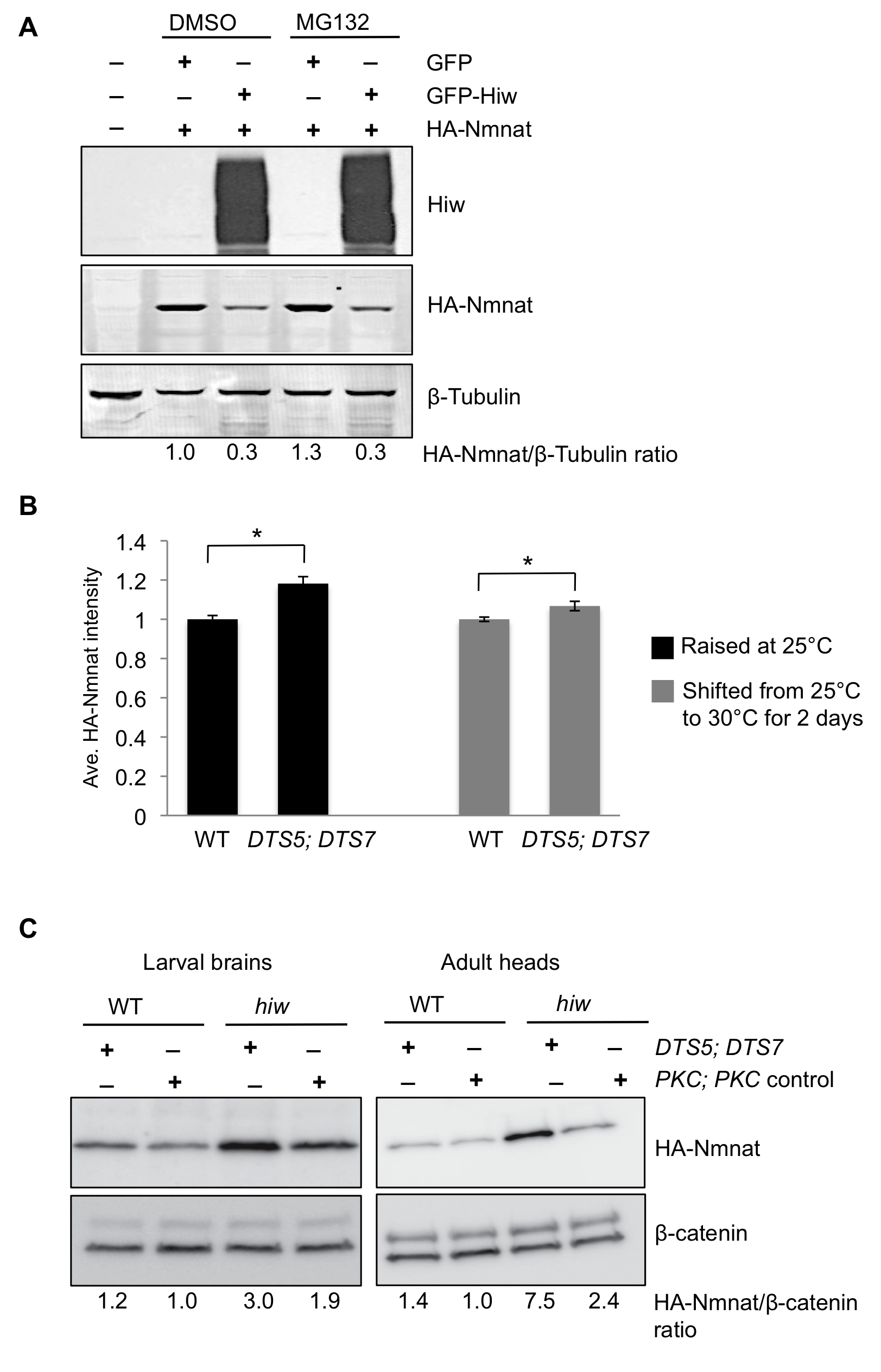

Supplement: Figure S4 — Hiw down-regulates Nmnat independently of the UPS. (A) The decrease in Nmnat levels promoted by Hiw induction in S2R+ cells was not diminished when the proteasome was inhibited. S2R+ cells co-transfected with pUAST-HA::Nmnat and pUAST-GFP or pUAST-GFP::Hiw, then were incubated with DMSO vehicle or 5 µM MG132 for 20 h. The levels of HA-Nmnat are compared by Western blotting for the HA epitope. Relative levels compared to the β-tubulin standard were measured on the LiCor Odyssey system. Similar results were observed for additional concentrations and time points (25 µM MG132 for 6 h, and 5 µM MG132 for 12 h, unpublished data). (B) Inhibition of the proteasome in sensory neurons only modestly changes HA-Nmnat levels. Average HA-Nmnat intensity in ppk sensory neuron axon terminals was compared between wild-type (UAS-HA::nmnat/+; ppk-Gal4,UAS-mCD8::RFP/+), and animals co-expressing DTS5 and DTS7 to inhibit the proteasome (UAS-HA::nmnat/UAS-DTS5; ppk-Gal4,UAS-mCD8::RFP/UAS-DTS7). Data are shown for two conditions: flies raised continuously at 25°C, and flies raised at 25°C, then shifted to 30°C for 2 d. Error bars represent standard error. *p<0.05. (C) Hiw and the UPS may influence Nmnat levels cooperatively. Total protein from third instar larval brains or young adult heads processed for Western blot from animals co-expressing DTS5 and DTS7 [45] to inhibit the proteasome (raised at 25°C), and compared to animals co-expressing two control UAS-PKC transgenes. Relative levels of HA-Nmnat protein, compared to the β-catenin standard, were measured on the LiCor Odyssey system. Combination of the hiw mutation with inhibition of the proteasome leads to much higher levels of HA-Nmnat, suggesting that Hiw and the UPS may potentially influence HA-Nmnat independently, rather than through the same pathway. Error bars represent standard error; *p<0.05 in t-test. (TIF) [file pbio.1001440.s004.tif]

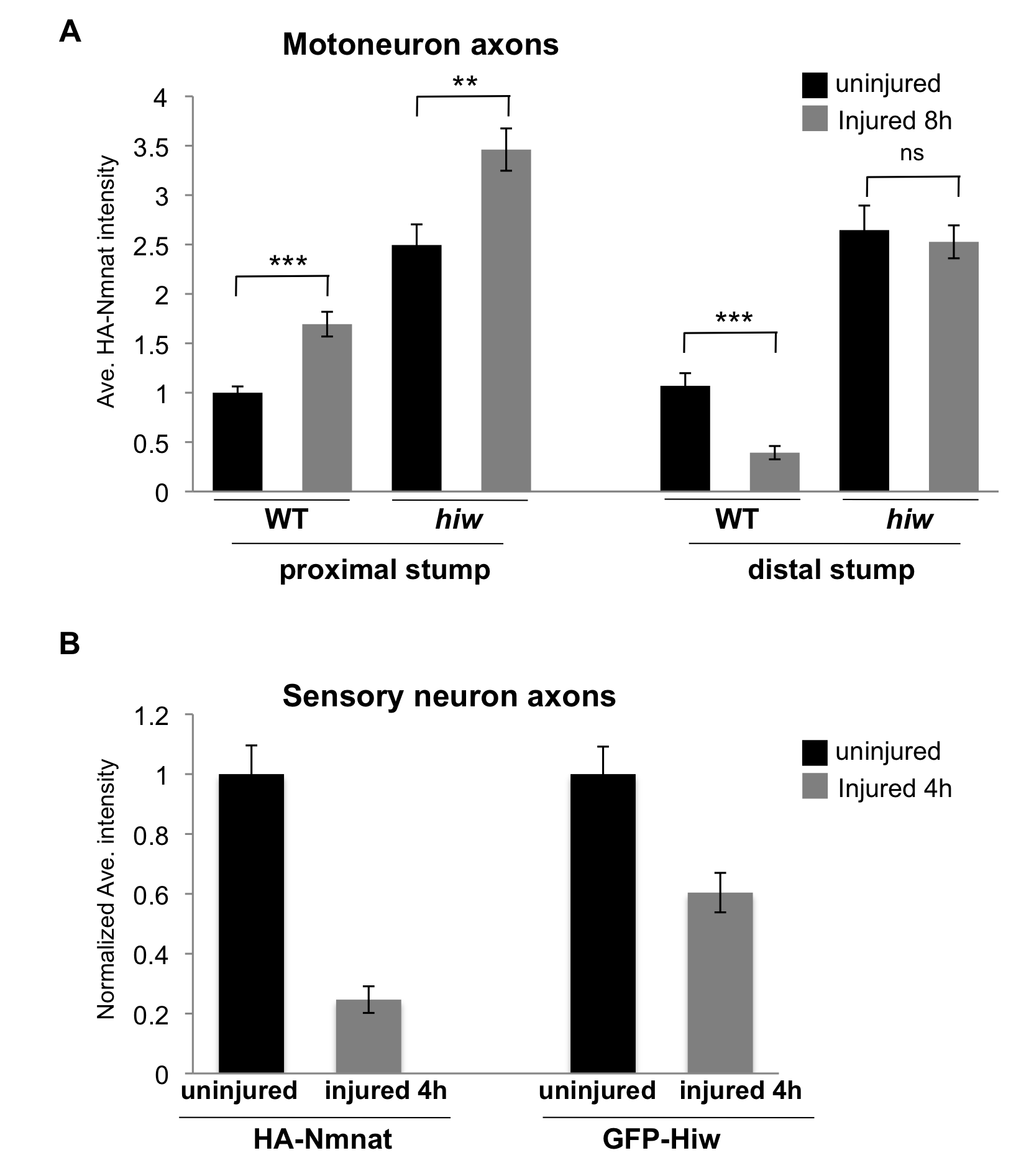

Supplement: Figure S5 — Changes in HA-Nmnat protein level in proximal and distal stumps, contrasted with GFP-Hiw after injury. (A) Quantification of average HA-Nmnat intensity in motoneurons axons for wild-type (OK6-Gal4/UAS-HA::nmnat) or hiw mutants (hiwΔN;OK6-Gal4/UAS-HA::nmnat) before (black) or 8 h after (gray) injury. HA-Nmnat levels in both proximal and distal axons were quantified and normalized to the average HA-Nmnat intensity in uninjured WT animals as described in Materials and Methods. Injury induces an increase of HA-Nmnat in the proximal stump in both wild-type and hiw mutant backgrounds. However, in the distal stump, the levels of HA-Nmnat reduced by 60.7% within 8 h in WT animals, but remained constant in hiw mutants. (B) Quantification of average HA-Nmnat and GFP-Hiw intensity in axon terminals of ppk-Gal4,UAS-mCD8::RFP labeled sensory neurons before or 4 h after injury. Error bars represent standard error; **p<0.01; ***p<0.001; ns, not significant, p>0.05 in t-test. (TIF) [file pbio.1001440.s005.tif]
